# AI‐Driven Acceleration of Fluorescence Probe Discovery

**DOI:** 10.1002/advs.202515604

**Published:** 2025-12-24

**Authors:** Xuefeng Jiang, Yanbo Li, Xue Tian, Sen Yang, Ruina Luo, Cenxing Zhou, Yuxuan Liu, Jingying Hu, Sen Feng, Lu Gan, Chongzhao Ran, Kun‐Hsing Yu, Junhan Zhao, Xiao Han, Xuan Zhai, Yuntao Jia, Jiapei Dai, Xiyue Wang, Biyue Zhu

**Affiliations:** ^1^ National Clinical Research Center for Children and Adolescents' Health and Diseases Children's Hospital of Chongqing Medical University Chongqing China; ^2^ Department of Radiation Oncology Stanford University School of Medicine Stanford California USA; ^3^ Athinoula A. Martinos Center For Biomedical Imaging Department of Radiology Harvard Medical School Massachusetts General Hospital Boston Massachusetts USA; ^4^ Department of Biomedical Informatics Harvard Medical School Boston Massachusetts USA; ^5^ Harvard Data Science Initiative Harvard University Cambridge Massachusetts USA; ^6^ College of Biomedical Engineering Sichuan University Chengdu Sichuan China; ^7^ Wuhan Institute for Neuroscience and Neuroengineering (WINN) Chinese Brain Bank Center (CBBC) College of Life Sciences South‐Central Minzu University Wuhan Hubei China

**Keywords:** fluorescence probes, in vivo imaging, molecular probes, optical imaging

## Abstract

Fluorescence imaging probes are indispensable tools for clinical navigation and preclinical research. However, the discovery of target‐specific probes is hampered by the scarcity of targetable fluorophore scaffolds, making the development process slow, costly, and heavily reliant on trial‐and‐error design. Here, we present a hybrid strategy that integrates AI with bioassays to accelerate the development of target‐specific fluorescent probes. We developed an AI model (PROBY) based on over one million molecule entries from nine datasets, capable of identifying fluorescent molecules and predicting seven key photophysical properties. Applying PROBY to a library of 26,416 target‐validated molecules, we identified thousands of candidates with both target affinity and favorable optical characteristics. Focusing on three clinically relevant targets (tau, BCL‐2, and TDP‐43), we validated AI‐identified candidates and discovered PE859, obatoclax, and B3, which supported applications in spectral analysis, drug screening, pathological labeling, cell imaging, and ex vivo tumor imaging. Guided by PROBY, we chemically modify PE859, yielding two optimized derivatives (859‐1 and 859‐2). With improved photophysical properties, 859‐2 enabled in vivo two‐photon imaging of tau pathology in transgenic mice. This hybrid AI‐bioassay strategy substantially broadens the accessible scaffold landscape for designing target‐specific fluorescence probes and provides a scalable, efficient, and cost‐effective framework for next‐generation probe discovery.

## Graphical abstract

We present PROBY, an AI model trained on large‐scale datasets to predict key photophysical properties and accelerate the discovery of target‐specific fluorescent probes. By screening a target‐annotated library, PROBY identifies candidate probes for diverse targets and could guide probe optimization, enabling a range of in vitro and in vivo imaging applications.

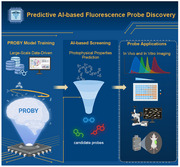

## 1 Introduction

Fluorescence imaging has emerged as an indispensable tool for visualizing biomolecular dynamics in real time, providing critical insights into disease mechanisms, diagnostics, and therapeutic interventions [1, 2, 3, 4]. Conventional approaches such as antibody labeling and protein tagging have laid the foundation for molecular imaging [5, 6]. More recently, small‐molecule fluorescent probes have attracted growing attention due to their operational simplicity, rapid labeling, and minimal perturbation of native biomolecular function, holding significant potential for clinical translation [7, 8, 9, 10]. Several fluorescence dyes and contrast agents, including indocyanine green (ICG), 5‐aminolevulinic acid (5‐ALA), hexaminolevulinate, and pafolacianine, have been approved by the FDA for clinical diagnosis and fluorescence image‐guided tumor surgery [9, 11]. However, most lack molecular specificity, which significantly limits their utility in target‐selective imaging [12]. This underscores the pressing need for next‐generation fluorescent probes with high target specificity and translational potential.

However, developing target‐specific fluorescent probes remains a significant challenge. The conventional strategies involve either modifying the fluorophore structure to enhance targeting capability, or conjugating the target moiety with a fluorophore [13, 14]. To meet the stringent requirements of an ideal fluorescence probe, such as excellent selectivity, stability, and fluorescence properties, probe development typically begins with a chemical scaffold and proceeds through multiple cycles of synthesis, characterization, and optimization [8, 12, 13, 14]. As a result, progress is constrained by the slow and iterative nature of probe development and achieving meaningful breakthroughs often takes years or even decades.

To facilitate fluorescence probe discovery, previous studies have sought to use computational methods, including HOMO‐LUMO gap calculations, time‐dependent density functional theory, and other quantum chemistry calculation methods, to predict fluorescence properties [15, 16]. These methodologies established the foundation for computation‐guided probe development. Despite these improvements, challenges remain in the high‐throughput screening of fluorescence probes with robust targeting capability [16, 17]. This highlights an unmet need for innovative strategies that can accelerate probe development while reducing time and cost.

Recent breakthroughs in artificial intelligence (AI) have revolutionized molecular property prediction through high‐throughput computational approaches [18, 19]. AI facilitates the exploration of chemical spaces orders of magnitude beyond traditional wet‐lab experimental capabilities, dramatically accelerating the identification of novel candidates [18, 20]. However, these AI‐driven strategies have largely focused on predicting bioactivity, toxicity, or pharmacokinetics, rather than on the design of functional fluorescence probes. Very recently, several AI models have been developed for fluorescence property prediction, including Gen‐DL [21], Fluor‐predictor [22], ESIPT predictor [23], ChemFLuo [24], and FLAME framework [25]. Despite these advances in high‐throughput prediction, current approaches remain limited by single‐task design, reliance on pre‐defined descriptors or small datasets. Furthermore, computational tools in this field are predominantly focused on optical property prediction. Although a few studies have reported in vitro validation data, none have been systematically validated or effectively applied to target‐specific probe discovery, particularly for in vivo imaging applications.

In this study, we present a deep learning–driven framework (PROBY) for the discovery of targeted fluorescent probes, integrating large‐scale computational screening with chemical and biological validation to enable target‐specific candidate probe discovery. We first curated a comprehensive dataset comprising over one million molecule entries with experimentally validated fluorescence properties, and used it to train PROBY models. Using PROBY, we conducted large‐scale virtual screening to identify probe candidates with both favorable fluorescence properties and target specificity. Furthermore, we selected three clinically relevant biomarkers, including B‐cell lymphoma 2 (BCL‐2), TAR DNA‐binding protein 43 (TDP‐43), and tau, for bioassay validation. Through spectral analysis, we identified obatoclax, B3, and PE859 as optimal fluorescent probes targeting BCL‐2, TDP‐43, and tau respectively, demonstrating their applications in drug screening, tissue labeling, cell imaging, and ex vivo tumor imaging. Focusing on PE859 as a representative candidate, we performed rational chemical modifications to optimize its optical properties, ultimately achieving two‐photon in vivo imaging of tau pathology in transgenic mouse models. Our work introduces an AI framework that overcomes prior limitations by establishing a paradigm shift from single‐task prediction to an integrated discovery pipeline. This pipeline combines tiered in silico screening with bioassay validation, consequently enabling the efficient discovery of target‐specific probes for a range of imaging applications.

## 2 Results

### 2.1 AI‐Guided Pipeline for Target‐Specific Fluorescence Probe Discovery

**FIGURE 1:**
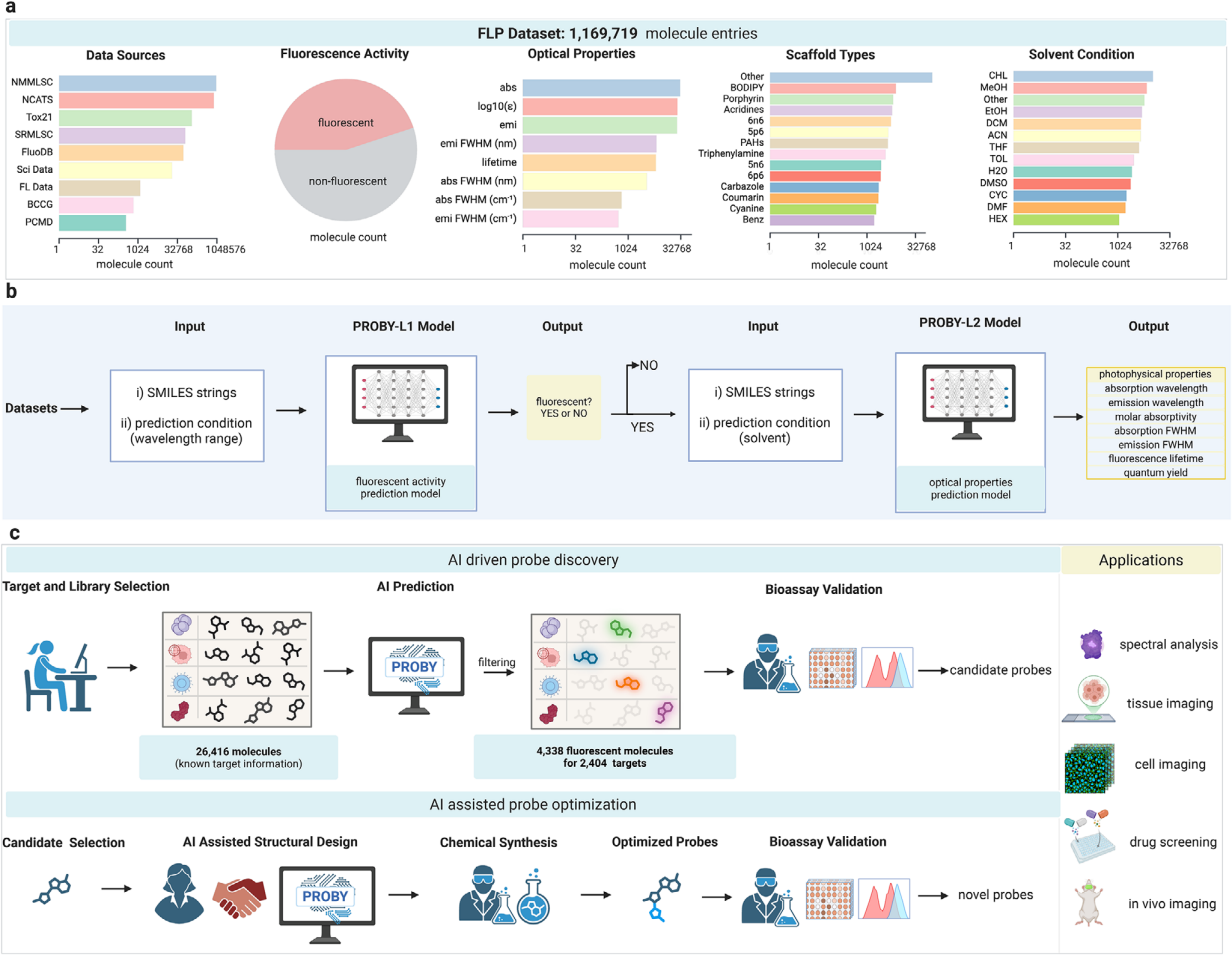
AI‐guided pipeline for target‐specific fluorescence probe discovery. (a) Composition of the collected molecular dataset. Photophysical data were compiled from 9 open‐access data sources, including NMMLSC, NCATS, Tox21, SRMLSC, FluoDB, Sci Data, SBCCG, PCMD, and another manually curated dataset (called FL dataset) sourced from literature searches via PubMed. These datasets comprise 1,169,719 molecular entries, covering both fluorescent and non‐fluorescent molecules (data are plotted on a log_2_ scale). The dataset encompasses diverse molecular scaffolds with seven key photophysical properties across more than 70 solvents, ensuring high chemical and optical diversity. (b) Two‐step deep learning pipeline for candidate probe identification. A high‐throughput screening workflow was developed comprising two deep learning models based on a message‐passing neural network architecture. PROBY‐L1 is a binary classification model developed on 983,790 molecule entries to predict fluorescence activity across specified wavelength condition. PROBY‐L2 is a regression model developed on curated datasets to predict key photophysical properties under diverse solvent conditions. The datasets include 31,358 molecules for absorption wavelength, 25,575 for emission wavelength, 3,487 for absorption full width at half maximum (FWHM), 6,598 for emission FWHM, 26,274 for molar absorptivity, 19,582 for photoluminescence quantum yield (PLQY), and 6,360 for fluorescence lifetime. SMILES strings, along with prediction conditions (wavelength range for PROBY‐L1 and solvent information for PROBY‐L2) were used as inputs. Non‐fluorescent molecules are filtered out by PROBY‐L1, while PROBY‐ L2 performs detailed optical property prediction for remaining candidates, enabling both efficient screening and refined probe selection. c. Overview of the AI‐guided probe discovery and optimization workflow. A library of molecules with known target affinity are screened using two‐step deep learning pipeline to identify candidates with favorable fluorescence properties. Selected probes undergo bioassay validations. Promising leads are further optimized via AI‐assisted structural design, chemical synthesis, and bioassay evaluation. The final probes are applicable to a range of biological applications, including spectral analysis, tissue and cell imaging, drug screening, and in vivo imaging.

To develop an AI‐driven probe discovery pipeline, we first collected a comprehensive dataset (named FLP) comprising 1,169,719 molecule entries from multiple public databases (NMMLSC, PCMD, NCATS, SRMLSC, Tox21, SBCCG from PubChem [26], Sci Data [27], and FluoDB [25]) and a manually curated dataset (FL dataset) from PubMed [26]. Each molecule was annotated with fluorescence activity (fluorescent or non‐fluorescent) and, where available, with photophysical properties encompassing absorption/emission wavelengths, molar absorptivity, full width at half maximum (FWHM), fluorescence lifetime, and photoluminescence quantum yield (PLQY) across over 70 solvent environments. This dataset provides high chemical and experimental diversity for model development (Figure 1a).

Next, we developed a two‐step high‐throughput screening pipeline for identifying candidate probes, called PROBY. PROBY comprises two deep learning models based on a message‐passing neural network architecture [28]. The first model, PROBY‐L1, is a fluorescence activity classifier capable of rapidly filtering out non‐fluorescent molecules. The second model, PROBY‐L2 is a regression model to predict key photophysical properties under diverse solvent conditions. This two‐tiered design enables both rapid screening and fine‐grained probe selection (Figure 1b).

Furthermore, we constructed a target‐specific fluorescence probe discovery pipeline that integrates large‐scale virtual screening with bioassay validation to identify and optimize fluorescent probes for diverse biological targets (Figure 1c). We used 26,416 small molecules from the TargetMol library, each with validated affinity for a specific target. These molecules were encoded as SMILES strings and screened by the PROBY models, leading to the discovery of thousands of fluorescent molecules targeting diverse biomarkers (Figure 1c). We further validated the AI‐identified molecules through bioassays, including fluorescence spectral scanning, binding assays, selectivity tests, and imaging performance evaluation. Furthermore, we chemically modified the candidate probes guided by PROBY predictions, resulting in novel probes with optimized photophysical properties (Figure 1c).

This hybrid AI‐bioassay workflow enabled rapid, target‐specific identification of fluorescent probes and demonstrated generalizability across targets. The resulting probes were applied in various preclinical studies, including pathological staining, cell imaging, and in vivo imaging, validating the translational potential of our pipeline.

### 2.2 Predictive Performance of PROBY and High‐Throughput Candidate Probe Screening

PROBY enables rapid and accurate detection of fluorescent molecules and predicts their photophysical properties, supporting high‐throughput screening of target‐specific fluorescence probe candidates.

**FIGURE 2:**
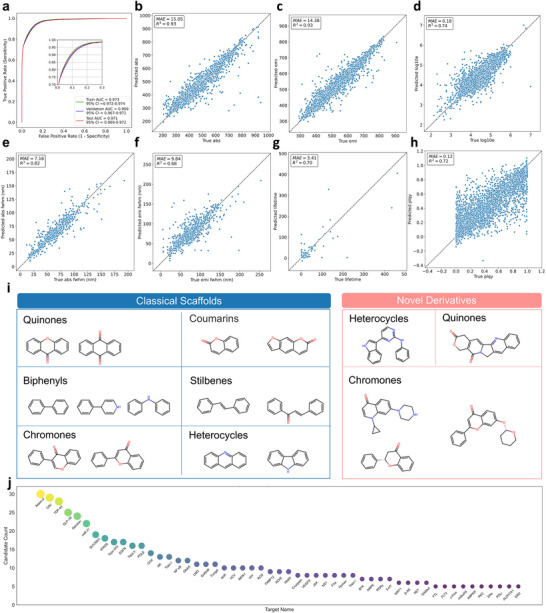
Prediction performance of PROBY and high‐throughput discovery of candidate probes. (a) Performance of PROBY‐L1. PROBY‐L1 yielded an area under the curve (AUC) of 0.971 on test datasets (n = 196,761), indicating strong predictive power for fluorescence activity. b,c. Prediction of absorption and emission wavelengths by PROBY‐L2. Parity plots of test datasets for the predicted versus experimental absorption (b, *n* = 6,272) and emission (c, *n* = 5,115) wavelengths. The model achieved *R* ^2^ values of 0.93 for both absorption and emission wavelengths. (d–f) Prediction of molar absorptivity and spectral width by PROBY‐L2. Parity plots of test datasets for molar absorptivity (log ɛ, mol^−1^⋅dm3⋅cm^−1^) (d, *n* = 5,255), absorption FWHM (e, *n* = 698), and emission FWHM (f, *n* = 1,320). PROBY‐L2 showed *R^2^* values of 0.74, 0.82, and 0.68, respectively. (g,h) Prediction of fluorescence lifetime and PLQY by PROBY‐L2. Parity plots of test datasets for lifetime (g, *n* = 1,272) and PLQY (h, *n* = 3,917). PROBY‐L2 showed *R* ^2^ values of 0.70 and 0.72, respectively. (i) Bemis–Murcko scaffolds of fluorescent molecules identified by PROBY. Scaffolds were extracted from valid SMILES using RDKit, encompassing both classical fluorescent scaffolds and novel fluorescent derivatives, all conforming to fundamental chemical principles. (j) Counts of candidate probes for representative targets discovered by PROBY. PROBY identified multiple probe candidates targeting diverse biomarkers, providing key chemical scaffolds for novel probe development.

In the binary classification task distinguishing fluorescent from non‐fluorescent molecules, we utilized the Area Under the ROC Curve (AUC) to evaluate the model. This metric reflects ranking ability, where 0.5 denotes random guessing and 1.0 represents perfect classification. PROBY‐L1 achieved an AUC of 0.969 (95% CI: 0.967–0.971) on the validation set (*n* = 98,380) and 0.971 (95% CI: 0.969–0.972) on the test set (*n* = 196,761, Figure 2a). In addition, it enables millisecond‐level inference with an average prediction time of 0.99 ms per molecule on a CPU.

For photophysical property prediction, PROBY‐L2 was evaluated across seven tasks using both validation (Extended Data Figure 1) and test datasets (Figure 2b–h). We utilized the coefficient of determination (*R^2^*) and the Mean Absolute Error (MAE) to evaluate the model. The *R^2^* metric measures the proportion of variance in the experimental data explained by the model, where a value of 1 indicates perfect prediction and 0 indicates no linear correlation. The MAE quantifies the average magnitude of prediction errors, with values closer to 0 signifying higher accuracy.

For absorption wavelength, the model achieved an *R* ^2^ of 0.94 with a mean absolute error (MAE) of 14.26 nm on the validation set, and an *R^2^* of 0.93 (MAE: 15.05 nm) on the test set. For emission wavelength, PROBY‐L2 yielded an *R^2^* of 0.94 (MAE: 13.51 nm) on the validation set and 0.93 (MAE: 14.38 nm) on the test set. In molar absorptivity (log10 *ε*_max_) prediction, the model reached an *R^2^* of 0.74 with MAEs of 0.17 and 0.18 on the validation and test sets, respectively. This high accuracy indicates that the model successfully captures structure‐property relationships governing electronic transitions, including bathochromic shifts induced by extended conjugation and solvatochromic behavior arising from differential stabilization of the ground and excited states in polar solvents.

For absorption FWHM, PROBY‐L2 achieved *R^2^* values of 0.79 (MAE: 6.90 nm) on the validation set and 0.82 (MAE: 7.16 nm) on the test set. For emission FWHM, the model yielded *R^2^* values of 0.76 (MAE: 9.26 nm) and 0.68 (MAE: 9.84 nm) for the validation and test sets, respectively. These results demonstrate that PROBY‐L2 effectively encodes the relationship between spectral linewidth and molecular flexibility.

In fluorescence lifetime prediction, PROBY‐L2 attained an *R^2^* of 0.67 (MAE: 3.45 ns) on the validation set and 0.70 (MAE: 3.41 ns) on the test set. For quantum yield (PLQY) prediction, the model achieved *R^2^* values of 0.71 (MAE: 0.12) on the validation set and 0.72 (MAE: 0.12) on the test set. These results reveal model's proficiency in characterizing radiative and non‐radiative decay processes, enabling the prediction of fluorescence efficiency and dynamics.

Fluorogenic properties are vital for target‐specific imaging. Therefore, we established predictive capability for this characteristic using a curated dataset of turn‐on and non‐turn‐on molecules from PubChem. The model showed an ROC‐AUC of 0.73, indicating its potential for predicting turn‐on capability of molecules (Extended Data Figure S2a).

PROBY‐L2 also supports high‐throughput inference with an average prediction time of 4.15 ms per molecule on a CPU.

Taken together, the model's high fidelity across these diverse properties confirms that the model successfully developed structure‐property relationships that align with established physico‐chemical principles.

Using the developed PROBY models, we conducted an independent screening of 26,416 small molecules from the TargetMol library, identifying 4,338 candidate molecules with predicted intrinsic fluorescence. To identify key chemical scaffolds among the candidate probes, Bemis–Murcko scaffolds were extracted from valid SMILES using RDKit. The resulting scaffolds encompassed both classical fluorescent scaffolds and novel fluorescent structures, all conforming to fundamental chemical principles (Figure 2i). These AI‐selected candidate probes were identified for 2,404 targets, including oncology, neuroscience, immunology, and metabolism (Figure 2j). These results provide a rich scaffold pool for further novel probe development. This framework substantially expands the accessible chemical space for probe design and offers a scalable strategy for accelerating target‐specific fluorescence probe discovery.

### 2.3 Photophysical Validation of AI‐Identified Probes

To demonstrate the applicability of our AI‐guided approach for fluorescent probe discovery, we selected three representative disease‐related targets for bioassay validation: tau [29], TDP‐43 [30, 31], and BCL‐2 [32, 33]. These targets are clinically implicated in neurodegenerative diseases and cancer, and their toxic forms serve as crucial biomarkers for etiology studies, early diagnosis, drug discovery, and personalized therapy. Specifically, tau aggregates drive Alzheimer's disease (AD), progressive supranuclear palsy, and Pick's disease [29]; TDP‐43 aggregates are hallmarks of amyotrophic lateral sclerosis (ALS) and frontotemporal lobar degeneration [30, 31]; BCL‐2 overexpression is oncogenic across diverse malignancies [32, 33]. These targets were chosen to establish a proof‐of‐concept framework for validating and optimizing probes identified by our model.

**FIGURE 3:**
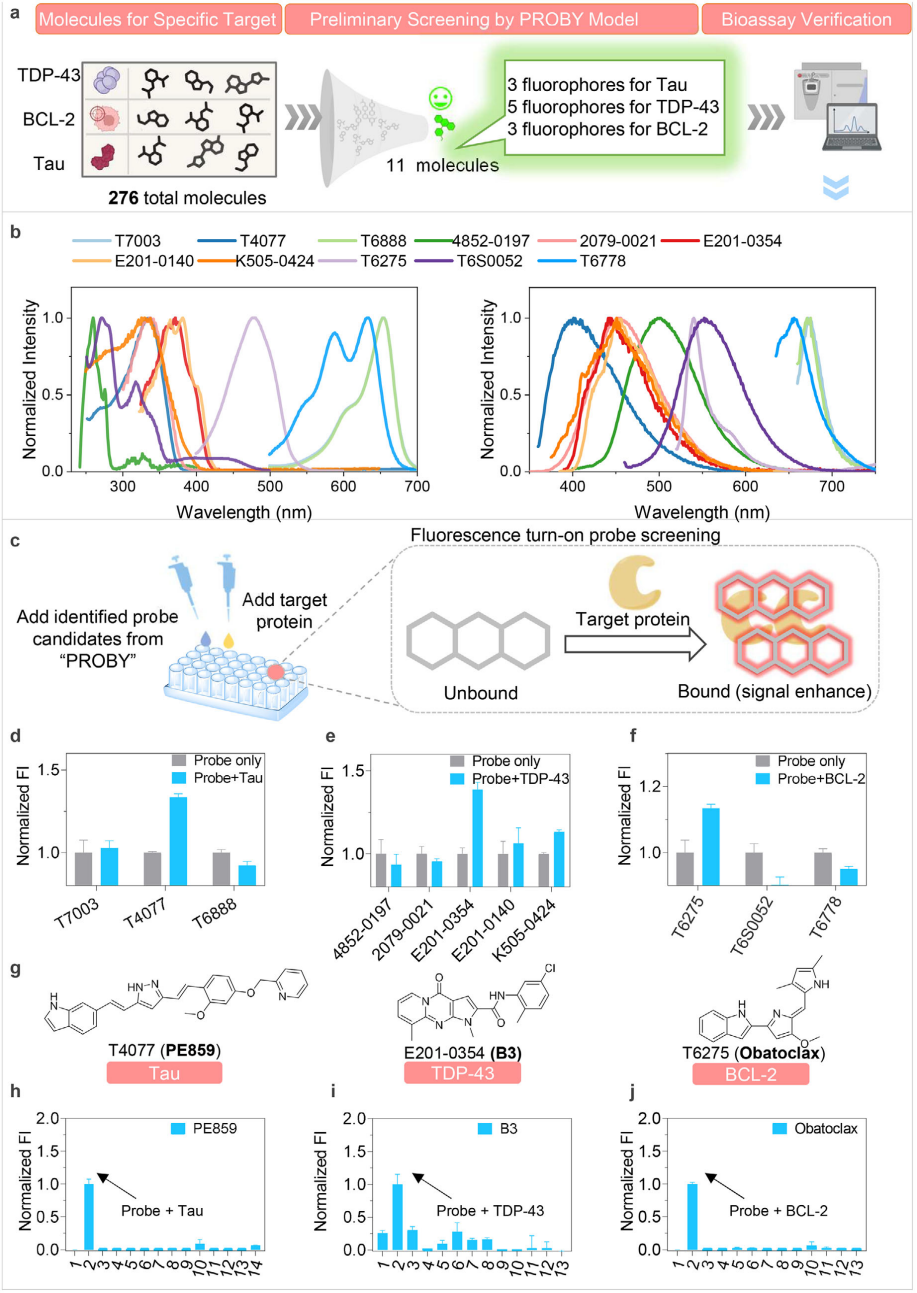
Photophysical validation of AI‐Identified probes. (a) Schematic overview of the AI‐driven discovery workflow for probes targeting tau, TDP‐43, and BCL‐2. 11 candidate molecules were identified by PROBY and subjected to further photophysical verification. (b) Verification of AI‐identified molecules by spectral assays. The absorption and emission spectra of all 11 molecules (10 µM) showed strong fluorescence in solvent of acetonitrile, confirming the robust predictive capability of PROBY. (c) Diagram of fluorescence turn‐on screening assays. To enhance imaging performance, we specifically screened fluorescent turn‐on probes. Candidate molecules (2 µM) were incubated with target protein or PBS control (1 h, room temperature) and fluorescence intensity was measured. (d–f) Fluorescence turn‐on screening results. Optimal probes were selected based on maximal intensity increase upon binding with tau aggregates (d), TDP‐43 aggregates (e), and BCL‐2 (f), The data are the mean ± S.E.M., *n* = 3. (g) Chemical structures of optimal candidate probes (PE859 for tau, B3 for TDP‐43, and obatoclax for BCL‐2). (h–j) Selectivity assessment of candidate fluorescent probes. PE859, B3, or obatoclax was mixed with various analytes (1. probe only, 2. tau aggregates or TDP‐43 aggregates or BCL‐2, 3. GSH, 4. Cys, 5. Glu, 6. Gly, 7.Na^+,^ 8. Fe^2+^, 9. Al^3+^, 10. K^+^, 11. Zn^2+^, 12. Cu^2+^, 13. Ca^2+^, 14. Aβ aggregates. The changes in fluorescence intensity were recorded and normalized to control (probe‐only), demonstrating exceptional target selectivity for all candidate probes, The data are the mean ± S.E.M., *n* = 5.

After the two‐step screening, we identified 3, 5, and 3 fluorescent candidates for tau, TDP‐43, and BCL‐2, respectively (Figure 3a). To evaluate the AI‐identified probes, we conducted spectral scanning assays and compared the experimentally measured photophysical properties with model predictions. All 11 molecules exhibited strong fluorescence signals, with absorption and emission wavelengths closely matching the model‐predicted values (Figure 3b, Extended Data Table S1). For example, the TDP‐43‐targeting molecule B3 was predicted with absorption and emission peaks at 370 and 437 nm (in acetonitrile), aligning with the spectral tested values of 370 and 443 nm (Extended Data Table S1). These results demonstrate the reliability of the AI‐based predictions. Furthermore, while the results reveal good agreement for extinction coefficients for most molecules, they highlight fluorescence quantum yield as a key challenge for prediction. A similar limitation is observed in other state‐of‐the‐art approaches, which may attributable to the scarcity of standardized and high‐quality quantum yield data for training (Extended Data Table S2).

The fluorogenic prediction by our model indicated that several of the 11 molecules possess promising turn‐on potential (Extended Data Figure S2b). Based on the 11 selected fluorescent molecules, we performed plate‐based fluorescence assays to identify optimal probes for tau, TDP‐43, and BCL‐2 (Figure 3c). Candidate probes were screened to identify molecules that could increase their fluorescence intensity when binding to the target proteins tau, TDP‐43, or BCL‐2, a property known as the fluorescence turn‐on effect, which enables more specific imaging [34, 35]. Among the tested candidates, T4077 (PE859), E201‐0354 (B3), and T6275 (obatoclax) demonstrated the most significant fluorescence enhancement in the presence of their respective targets (Figure 3d–f). Notably, these findings are consistent with previous reports identifying these molecules as high‐affinity binders of tau (PE859, IC_50_ = 0.66 µm), TDP‐43 (B3, IC_50_ = 1.122 µm), and BCL‐2 (obatoclax, *K*_i_ = 220 nm) [36, 37, 38].

The chemical structures and optical spectra of PE859, B3, and obatoclax are shown in Figure 3g. All three molecules contain conjugated structures with benzoheterocyclic rings and multiple double bonds, serving as promising scaffolds for fluorescence turn‐on responses [39, 40] (Extended Data Figure S3). Selectivity analysis revealed that 3 molecules exhibited target‐specific fluorescence turn‐on responses with negligible interference from other biomolecules, including trypsin, human serum albumin (HSA), lysozyme, and bovine serum albumin (BSA) (Figure 3h–j; Extended Data Figure S4 and Table S3).

Furthermore, to explore the model's capability for screening novel structures, we applied PROBY to screen the ChemDiv library and successfully identified a previously unreported compound. The experimental measurements of its photophysical properties showed an agreement with the model's predictions (Extended Data Figure S5).

Collectively, these findings demonstrate both the reliability of our AI model and its successful application in discovering PE859, B3, and obatoclax as optimal candidate fluorescence probes for tau, TDP‐43, and BCL‐ 2, respectively.

### 2.4 In Vitro Applications of Validated Target‐Specific Probes

Following the identification of the three optimal candidate probes (PE859, B3, and Obatoclax), we conducted bioassays to further validate the effectiveness of the AI‐identified probes and explore their potential for preclinical applications.

**FIGURE 4:**
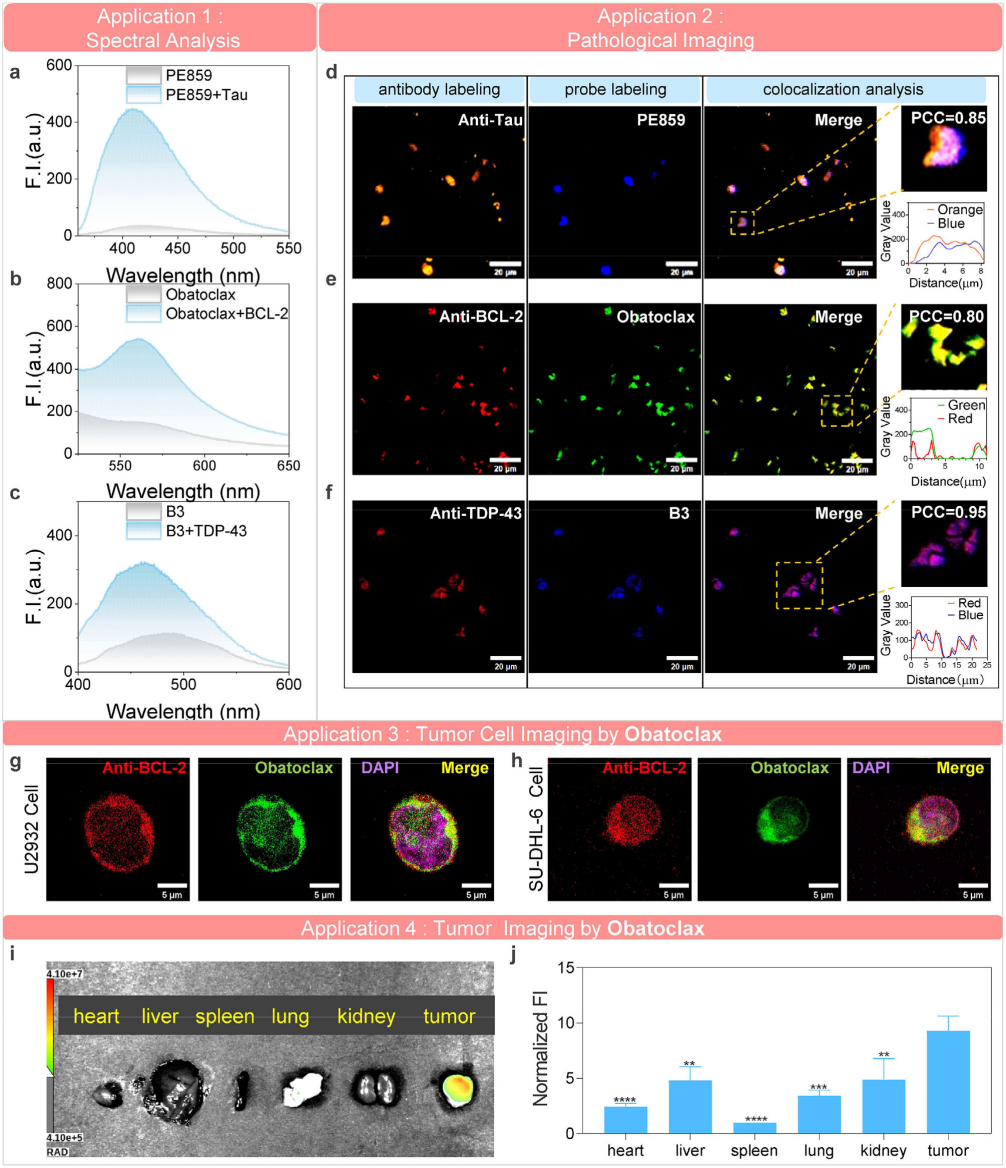
In vitro applications of validated probes. (a–c) Spectral analysis applications. The mixture of fluorescence probes with corresponding biomarkers revealed fluorescence enhancement with characteristic spectral shifts, indicating detection capability of probes in solution. (d–f) Pathological imaging applications. Fluorescence microscopy images of tissue sections from Alzheimer's disease brain, amyotrophic lateral sclerosis brain, and neuroblastoma tumor, stained with PE859, B3, and obatoclax, respectively. Co‐staining with target‐specific antibodies displayed good colocalization, confirming the probes’ pathological labeling capability. (g,h) Cell imaging applications of obatoclax. BCL‐2‐overexpressing U2932 (g) and SU‐DHL‐6 (h) cells were stained with obatoclax and imaged by confocal microscopy. Anti‐BCL‐2 antibody co‐staining showed good colocalization, confirming cellular labeling capability of obatoclax for BCL‐2. (i,j) Ex vivo fluorescence imaging of obatoclax distribution in SU‐DHL‐6 lymphoma xenografts (*n* = 3). Representative images (i) and quantitative analysis (j) of tumors and major organs (heart, liver, spleen, lung, and kidney) 4 h post‐injection (10 mg/kg, i.v.) demonstrate selective accumulation in BCL‐2‐overexpressing tumors versus normal tissues. The data are the mean ± S.E.M., *n* = 3. Statistical significance was assessed using a one‐way ANOVA followed by Dunnett's multiple comparisons test versus the tumor group, ****p < 0.0001, ***p < 0.001, **p < 0.01. These results support obatoclax as a candidate fluorescent scaffold for tumor imaging.

First, we performed spectral assays to evaluate the fluorescence changes of the three probes upon target binding (Figure 4a–c). The results revealed distinct target‐induced fluorescence enhancement and wavelength shift for all probes. PE859 showed strong fluorescence enhancement with a 10 nm blue shift in emission maximum upon binding to tau aggregates. Obatoclax exhibited a 7 nm blue shift and increased fluorescence intensity in the presence of BCL‐2. B3 displayed the most pronounced wavelength change, with a 30 nm blue shift and fluorescence enhancement upon interaction with TDP‐43 aggregates. These results demonstrate the applications of probes for spectral detection and biochemical environmental analysis of their respective targets.

To evaluate the labeling performance of three probes in pathological tissues, we utilized patient‐derived brain sections from AD and ALS, along with neuroblastoma tumor sections, with each section used to assess one of the probe–target pairs. Successful target labelings of probes were confirmed by co‐staining with the corresponding antibodies and performing colocalization analysis. PE859 fluorescently labeled tau aggregates in AD brain sections, showing good spatial overlap with anti‐tau antibody staining (Pearson Correlation Coefficient, PCC = 0.85 for region of interest) (Figure 4d). Obatoclax exhibited good colocalization with anti‐BCL‐2 antibody in neuroblastoma tumor tissue sections (PCC = 0.80) (Figure 4e). B3 successfully labeled TDP‐43 aggregates in brain sections from an ALS patient, with a PCC of 0.95 relative to anti‐TDP‐43 antibody staining (Figure 4f). Notably, while antibody staining requires multiple incubation and washing steps that typically take 1 day to complete, our probes enable target protein labeling within 10 min. These results demonstrate the probes’ capability for rapid and target‐specific fluorescence labeling in clinically derived pathological tissues.

Furthermore, we selected obatoclax as a representative probe for cellular imaging studies, given the established utility of BCL‐2 visualization in apoptotic cell lines for preclinical research [41]. Immunofluorescence co‐staining with anti‐BCL‐2 antibodies confirmed specific target labeling in cells. As shown in Figure 4g, obatoclax effectively detected BCL‐2 in overexpressing cell lines (U2932 and SU‐DHL‐6). In addition, we established a mouse tumor model using SU‐DHL‐6 cells and administered obatoclax via tail vein injection. At 4 h post‐injection, obatoclax showed higher accumulation in tumors compared to other organs, demonstrating its potential as a candidate scaffold for tumor visualization (Figure 4i,j).

In addition, we employed B3 as a representative fluorescence reporter for drug screening, motivated by the emerging role of TDP‐43 as a critical therapeutic target in ALS and the urgent need for novel TDP‐43‐ targeted therapeutics [31]. We developed a B3‐based assay to identify inhibitors (drug candidates) that bind to TDP‐43, where target binding is indicated by a reduction in B3 fluorescence intensity. To enhance screening efficiency, we first performed computational pre‐screening and identified five candidates (Extended Data Figures S6 and S7, and Extended Data Table S4), which were then evaluated using B3‐based assay (Extended Data Figure S8a,b). Among these, A5 exhibited the strongest fluorescence inhibition of B3 (Extended Data Figure S8c). In addition, we conducted conventional Thioflavin T (ThT) assay and microscale thermophoresis (MST) measurement to validate the drug candidate A5 identified by B3. ThT assays confirmed the inhibitory effect of A5 on TDP‐43 aggregation, as evidenced by decreased ThT fluorescence signal (Extended Data Figure S8d). MST further confirmed the direct binding of A5 and TDP‐43, showing a dissociation constant *K*_d_ value of 445 ± 141 nm (Extended Data Figure S8e). These results indicated the utility of B3 in drug screening and led to the identification of A5 as a novel TDP‐43 inhibitor.

These experimental results further confirmed that the AI‐guided discovery workflow can effectively identify candidate fluorescence probes for various molecular targets. The three validated probes represent versatile tools for preclinical applications in neuroscience and oncology, including spectral analysis, pathological labeling, cellular imaging, tumor imaging, and drug screening.

### 2.5 AI‐Guided Design of Novel Probes and Bioassay Validations

**FIGURE 5:**
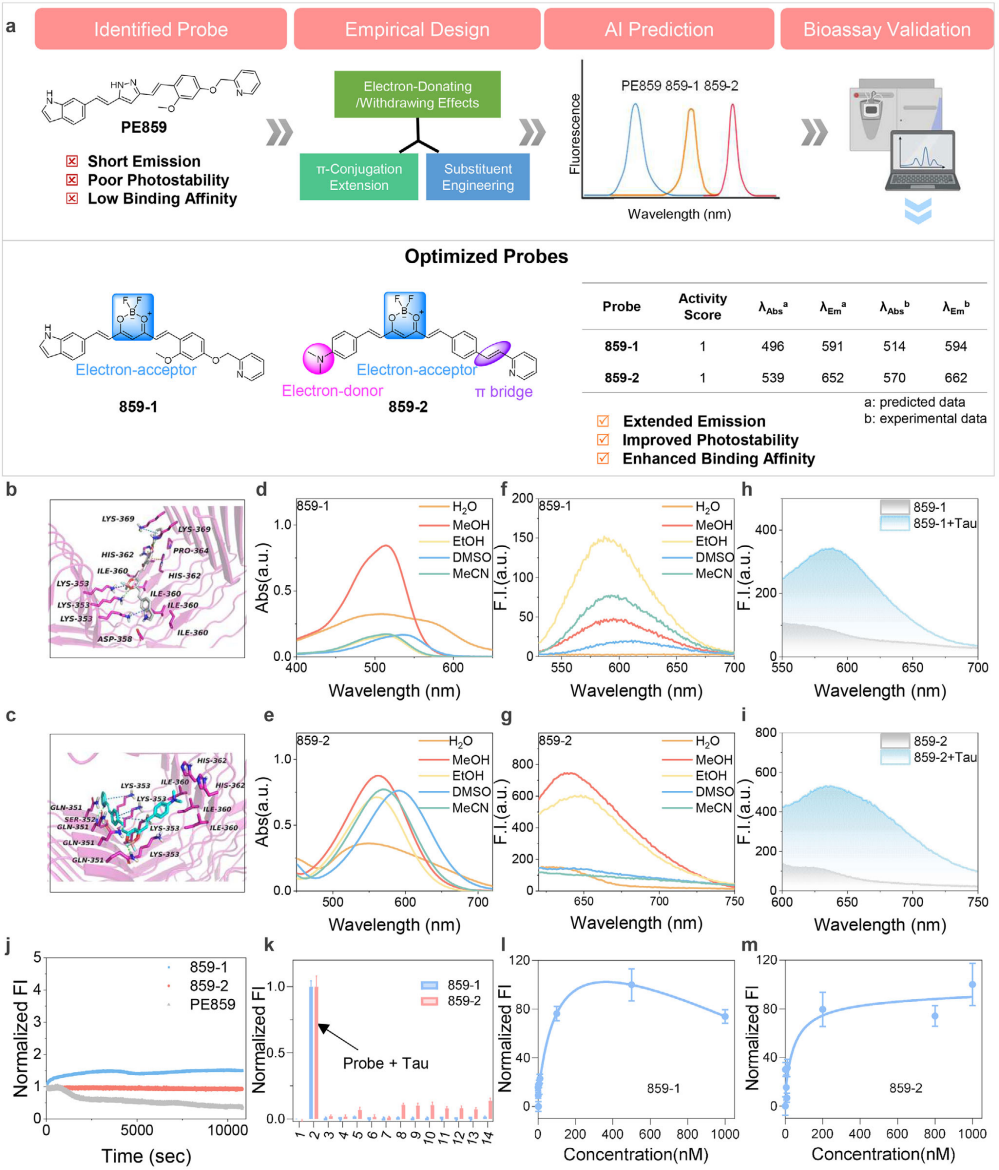
Probe optimization and photophysical validation. (a) Diagram of the probe optimization workflow. PE859 was modified based on empirical design, AI prediction and photophysical validation, leading to the discovery of two optimized probes, 859‐1 and 859‐2. (b,c) Computational prediction of the targeting capabilities of 859‐1 (b) and 859‐2 (c). Docking studies indicated that both probes could bind to tau fibrils (PBD: 5O3L). (d–g) Absorption spectra (d,e) and emission spectra (f,g) of 859‐1 and 859‐2 in various organic solvents. The photophysical results were consistent with PROBY‐predicted optical properties, indicating optimized optical properties. (h,i) Fluorescence spectra of 859‐1 or 859‐2 (2 µM) with or without tau aggregates (4 µM). Both probes exhibited a fluorescence turn‐on effect upon target binding. (j) Photostability studies of PE859, 859‐1, and 859‐2. Solutions of each probe (2 µM in PBS, DMSO/PBS = 1/9, v/v) were continuously irradiated on a fluorescence spectrometer, and the emission intensity was monitored over time. The optimized probe 859‐2 exhibits superior photostability compared to PE859. (k) Selectivity assessment of optimized probes. 859‐1, or 859‐2 was mixed with various analytes (1. probe only, 2. tau aggregates, 3. GSH, 4. Cys, 5. Glu, 6. Gly, 7. Na^+,^ 8. Fe^2+^, 9. Al^3+^, 10. K^+^, 11. Zn^2+^, 12. Cu^2+^, 13. Ca^2+^, 14. Aβ aggregates). The fluorescence intensities were recorded and normalized to the probe‐only control. Both probes showed excellent selectivity for tau aggregates. The data are the mean ± S.E.M., *n* = 3. (l,m) Binding affinities tests. Tau aggregates were titrated with 859‐1 (l) or 859‐2 (m) and the fluorescence intensities were recorded. The dissociation constant (*K*_d_) was calculated by plotting the intensity against probe concentration, showing higher binding affinity of 859‐2 (*K*_d_ = 54.8 nM) than 859‐1 (*K*_d_ = 216.8 nM). The data are the mean ± S.E.M., *n* = 3.

To extend beyond candidate identification, we established an AI‐guided workflow for the rational design of novel fluorescent probes (Figure 5a). PE859 showed target‐specific fluorescence turn‐on response, but its short emission wavelength of only 400 nm suffers from tissue autofluorescence interference and UV‐induced cytotoxicity. Thus, we used PE859 as an example for developing novel probes with optimized photophysical properties [42].

We empirically designed a series of derivatives based on a donor‐π‐acceptor (D‐π‐A) rationale and used PROBY models to predict their photophysical properties. Based on AI predicted results, two derivatives (859‐1 and 859‐2) ranked top with the highest activity score and substantially improved photophysical properties (Figure 5a). The first derivative, 859‐1, was generated by replacing the pyrazole moiety with a difluoroboronate moiety, a well‐established electron acceptor known to promote red‐shifted emission [43]. The second derivative, 859‐2, was designed by introducing an *N,N’*‐dimethylamino group, a strong red‐shift‐inducing substituent [44], at the para position of the benzene ring, and replacing ether linkages with carbon‐carbon double bonds to extend conjugated system and facilitate longer emission (Figure 5a).

We also combined molecular docking tools for computationally predicting the binding between designed derivatives and tau fibrils (PDB ID: 5O3L, Figure 5b,c). Both 859‐1 and 859‐2 show strong binding to tau fibrils through multiple interactions, including cation–π, *π*–*π* stacking, and hydrogen bonds with key residues (Lys353, His362), as supported by docking models (Figure 5b,c). These computational results suggested 859‐1 and 859‐2 as optimized tau‐targeting fluorescence probes.

After confirming the rationality of designed probes, we synthesized 859‐1 and 859‐2 via consecutive aldol condensation between aldehydes and an acetylacetone boron‐difluoride intermediate in acetonitrile [43, 45, 46]. With 859‐1 and 859‐2 in hand, we first tested their photophysical properties in various solvents. As expected, both 859‐1 and 859‐2 exhibited significantly longer absorption and emission wavelengths compared to PE859 (Figure 5d–g; Extended Data Table S5). In MeCN, 859‐1 exhibited an emission wavelength peak at 594 nm, showing a redshift of 192 nm compared to PE859 (Figure 5f; Extended Data Table S5). Similarly, 859‐2 displayed an emission wavelength peak at 662 nm, exhibiting a 260 nm redshift (Figure 5g; Extended Data Table S5). Furthermore, the fluorescence intensity of both probes was inversely correlated with solvent polarity, being strongest in low‐polarity environments (Figure 5f,g; Extended Data Table S5). In addition, the effect of viscosity on the fluorescence of the probes was examined in glycerol/DMSO mixtures across a glycerol fraction range of 0% to 90%. A significant increase in fluorescence intensity was observed for both probes with increasing viscosity (Extended Data Figure S9). This solvent‐dependent behavior aligns with typical D‐π‐A fluorophore characteristics, suggesting that 859‐1 and 859‐2 are environmentally sensitive. In addition, density functional theory (DFT) calculations of frontier molecular orbitals revealed that chemical modification reduced the highest occupied molecular orbital (HOMO)‐lowest unoccupied molecular orbital (LUMO) energy gap (Extended Data Figure S10), showing ∆*E* = 2.4939 and 2.3350 eV for 859‐1 and 859‐2, respectively. This decrease in energy gap explains the observed emission redshift, as smaller ∆*E* requires less energy for electronic transitions [47]. The agreement of results among AI prediction, DFT calculations, and bioassays validates AI‐guided probe optimization strategies. We next evaluated their fluorescence turn‐on response upon target binding. When incubated with tau aggregates, both 859‐1 and 859‐2 exhibited substantially increased fluorescence intensity, with emission redshifts of 6 nm and 4 nm, respectively (Figure 5h,i). These results demonstrate that the structural optimization of PE859 successfully extended emission wavelengths while retaining fluorescence turn‐on capability in response to tau.

We then assessed the photostability of the probes under continuous light exposure. 859‐2 demonstrated improved photostability compared to PE859. Notably, 859‐2 maintained stable fluorescence over a 3 h illumination period, while 859‐1 showed a 152% fluorescence intensity variation (Figure 5j), highlighting 859‐2's superior suitability for long‐term imaging applications.

To evaluate target selectivity, fluorescence assays were conducted in the presence of tau aggregates and various non‐specific analytes, including proteins, amino acids, and metal ions. Both 859‐1 and 859‐2 exhibited strong fluorescence signals responses with tau, but minimal response to other analytes (Figure 5k; Extended Data Figure S11). Furthermore, we determined the binding affinities of the probes with tau aggregates. Both probes demonstrated favorable binding affinity, with 859‐2 showing significantly higher affinity (*K*_d_ = 54.8 nm) compared to 859‐1 (*K*_d_ = 216.8 nm). These results are consistent with the molecular docking findings (Figure 5l,m; Extended Data Table S6).

Taken together, these results confirm the success of our structure‐guided optimization strategy. The optimized probes exhibit extended emission wavelengths, enhanced photostability, and high selectivity and affinity for tau aggregates, making them highly promising candidates for advanced bioimaging applications.

### 2.6 In Vitro and In Vivo Applications of Optimized Probes

Following the validation of the optimized probes in solution‐phase assays, we further assessed their effectiveness and translational potential through a series of preclinical imaging experiments, including pathological imaging, two‐photon in vivo imaging, and transparent tissue 3D imaging.

**FIGURE 6:**
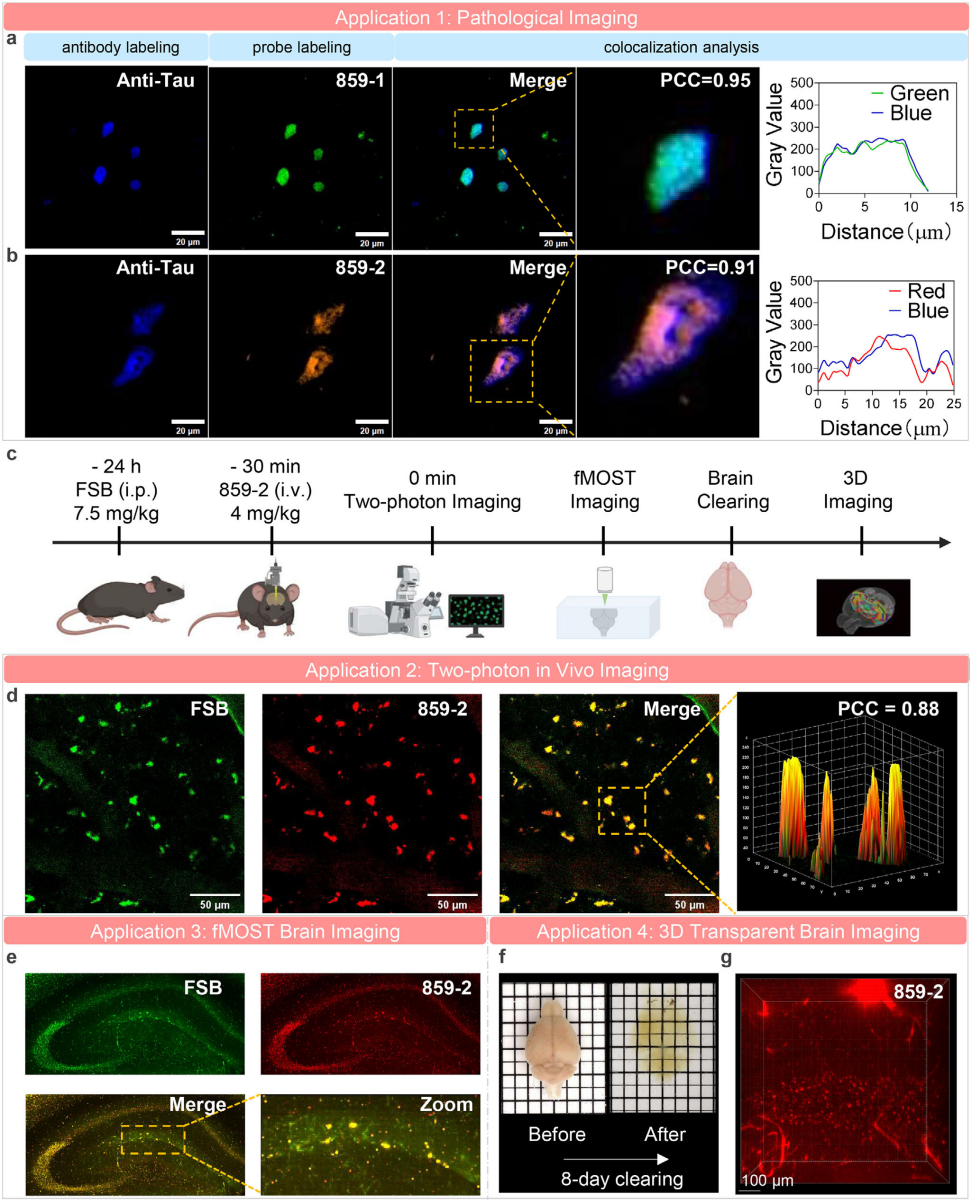
In vitro and in vivo applications of optimized probes. (a,b) Pathological imaging applications. Probes (a) 859‐1 and (b) 859‐2 selectively labeled tau deposits in Alzheimer's disease brain sections, as validated by anti‐tau antibody colocalization. (c) Schematic of in vivo imaging studies. Two‐photon microscopy and ex vivo 3D tissue imaging were performed in 9‐month‐old female P301S tau transgenic mice (*n* = 3) using probe 859‐2 to visualize tau aggregates in the brain. (d) Representative in vivo two‐photon fluorescence imaging data. Probe 859‐2 successfully labeled tau aggregates in the brain of transgenic mice, as confirmed by FSB (reported fluorescence dye for tau) staining and colocalization analysis. The representative boxed region was magnified, and the intensity profile and PCC (Pearson's correlation coefficient) were displayed. (e) Representative fMOST brain imaging of P310S transgenic mice. 859‐2 exhibited strong tau labeling, indicating it as a novel probe for fMOST imaging studies. (f,g) Representative ex vivo 3D transparent brain imaging results of transgenic P301S mice. Brain lipids were removed (f) by an 8‐day tissue clearing process. The exceptional stability of 859‐2 enabled strong tau‐labeling in cleared brain tissues (g), overcoming the quenching issues that limit most dyes during prolonged clearing processes.

We first performed pathological staining experiments to assess the ability of the optimized probes to label tau aggregates in patient‐derived brain tissue sections. Target‐specific labeling was confirmed via co‐staining with anti‐tau antibodies and colocalization analysis. Both 859‐1 and 859‐2 successfully labeled tau aggregates in AD brain tissues, with strong colocalization with antibody signals (PCC = 0.95 and 0.91, respectively) (Figure 6a,b). We further validated probe specificity by performing staining on tissues that endogenously lack the pathological targets. Our fluorophore produced only minimal background signal in these sections, as validated by co‐localization with a standard antibody. These data indicate the observed staining is target‐dependent (Extended Data Figure S12). Together, these findings verify the high selectivity of 859‐1 and 859‐2 for pathological tau aggregates.

We next performed in vivo two‐photon microscopy to evaluate the probe's capability for dynamic, high‐resolution imaging of tau aggregates under physiological conditions. Prior to imaging, we evaluated the blood‐brain barrier (BBB) permeability of 859‐2 to assess its potential for neuroimaging. Pharmacokinetic analysis in mice following intravenous administration revealed that the brain concentration of 859‐2 peaked at 15 min, with a brain‐to‐plasma ratio (Kp, brain) of 0.34, indicating BBB penetration (Extended Data Figure S13). Based on its superior photophysical properties, binding affinity, and photostability, 859‐2 was selected for live imaging in 9‐month‐old P301S tau transgenic mice, which exhibit abundant brain tau aggregates. The reference dye FSB was used as a control for validating tau‐specific labeling (Figure 6c). Prior to co‐imaging, we confirmed that FSB produced negligible fluorescence in the 859‐2 imaging channel (red channel, 1000 nm excitation) (Extended Data Figure S14a). Co‐imaging subsequently revealed that 859‐2 effectively labeled tau deposits in the red channel, with high spatial overlap with FSB fluorescence in the green channel (800 nm excitation) (Figure 6d). Furthermore, fluorescence micro‐optical section tomography (fMOST) was utilized for ex vivo brain imaging of P301S mice. 859‐2 showed substantial labeling of tau deposits in the hippocampus region confirmed by FSB staining (Figure 6e). These results indicated that 859‐2 enables dynamic in vivo visualization of tau pathology in transgenic mouse models.

Following in vivo two‐photon imaging, we evaluated 859‐2 for transparent tissue 3D imaging, which is an emerging technique for biomarker visualization that currently lacks stable probes resistant to prolonged tissue‐clearing processes [48,49]. Leveraging 859‐2's exceptional stability, we performed ex vivo tissue clearing on transgenic mouse brains after imaging (Figure 6c). After 8 days of clearing, lipids were removed for high‐resolution 3D imaging of transparent brains (Figure 6f). Consistent with in vivo two‐photon imaging results, 859‐2 robustly labeled tau aggregates (Figure 6g). In contrast, FSB signals were nearly abolished due to its inability to withstand the clearing process (Extended Data Figure S14b). These results demonstrate 859‐2's unique suitability for both in vitro and in vivo imaging of tau aggregates.

## 3 Conclusion

We developed the first AI‐driven framework (PROBY) for target‐specific fluorescent probe discovery based on the largest photophysical molecular dataset to date. Using PROBY, we identified a wide range of target‐specific probe candidates. Through combined bioassay validation, we successfully discovered probes for tau, BCL‐2 and TDP‐43 with applications ranging from molecular analysis to live imaging.

PROBY represents the first hybrid workflow that enables efficient identification of candidate probes across a wide range of targets, addressing a critical gap of current computational tools for efficient and target‐ specific fluorescent probe discovery. Specifically, the widely used TD‐DFT method provides accurate quantum chemical predictions but is limited by its high computational cost, typically requiring over 200 CPU hours per molecule [17, 25]. ABT‐MPNN, an atom‐bond Transformer‐based model, demonstrated strong prediction performance by integrating Transformer architectures with graph neural networks, but its attention mechanisms impose a speed penalty [25, 50]. More recently, FLAME achieves sub‐second predictions through efficient message‐passing architectures trained on 55,169 fluorophores [25], yet remains limited to optical property prediction without target engagement validation. To date, none of the computational methods have been systematically validated or effectively applied to target‐specific probe discovery, particularly for in vivo imaging applications. Our models support multi‐task prediction across seven key photophysical properties and achieve millisecond‐level inference speed. In an independent screening of 26,416 molecules from the TargetMol library, we successfully identified thousands of candidate fluorescent scaffolds associated with over 2000 targets across oncology, neuroscience, immunology, and other fields. These advances markedly expand the chemical diversity of fluorescent probe scaffolds and hold strong translational potential after further validation and optimization.

For bioassay validations, we validated novel probes targeting key biomarkers in oncology (BCL‐2) and neuroscience (TDP‐43 and tau) by using fluorescence spectroscopy and imaging experiments. In addition, we also provided a probe optimization paradigm from AI‐identified candidates and developed new tools for detecting the crucial biomarker of tau protein. We identified PE859 as a fluorescence candidate probe and optimized its structure to develop two new probes (more synthesis details in Extended Data Figures S15–S25) with optimized properties. These findings provide valuable tools for the study of etiology, as well as diagnostic and therapeutic development in neuroscience and oncology.

The implications of our strategy span from fundamental biology to translation applications. In preclinical research, our platform facilitates discovering probes for real‐time visualization of disease biomarkers, showing broad applications in mechanistic studies of pathogenesis and therapeutic evaluations [2, 13]. Moreover, our framework can be extended to support multiplexed imaging, single‐cell analysis, and high‐content screening [4, 13, 51]. For translation applications, PROBY empowers the development of both diagnostic and theranostic tools for pathological staining, biofluid‐based detection, and fluorescence imaging‐guided surgery [2, 3, 8, 9]. Notably, our strategy could address the critical need for target‐specific imaging in surgical navigation, with potential applications in precise tumor margin delineation, high‐resolution vascular mapping, and lymphatic system visualization, etc. Given fluorescence imaging is the most versatile and cost‐effective preclinical imaging modality, our platform could also facilitate development of multi‐modal imaging probes (e.g., fluorescence‐PET or fluorescence‐MRI probes).

Notably, this study only focuses on molecular repurposing, which could significantly accelerate probe development by leveraging pre‐validated properties, including target affinity, toxicity, and metabolic profiles. Having prioritized known molecules for initial validation, we now have a proven AI‐assisted discovery pipeline with the potential to identify probes for a broad range of targets. The next phase of chemical innovation will involve incorporating computational binding prediction and generative AI into molecular design, alongside developing new synthetic methods for probe modification. This is particularly impactful for difficult targets lacking candidate probes [11, 12], where conventional probe development is limited by low throughput and suboptimal specificity [15, 17, 52].

Here, we outline the main limitations of this study and discuss promising future research directions.: (1) While most probes demonstrated excellent specificity, B3 showed off‐target activation with HSA and lysozyme, highlighting a limitation of model‐predicted probes in complex biological environments. To enhance specificity, future work will refine our screening criteria to better balance fluorescence performance and specificity from the outset. Integrating in silico binding screening is also expected to guide the rational design of improved probes. (2) The prediction of fluorogenic behavior is pivotal for developing target‐specific fluorescent probes, yet it remains challenging due to limited experimental data and complex turn‐on mechanisms [14, 22]. Our preliminary fluorogenic prediction model achieved an AUC of 0.73 and correctly identified 7 out of 11 turn‐on probes in experimental validation. This demonstrates a promising foundation. Future efforts will focus on expanding the training set and incorporating advanced mechanistic descriptors to enhance generalizability and accuracy. (3) Although our model enables millisecond‐scale prediction of several photophysical properties, we observed some inconsistencies, particularly in quantum yield. This limitation is not unique to our model but is also present in other state‐of‐the‐art approaches, which may be due to the scarcity of standardized and high‐quality quantum yield data for training. Future efforts in high‐quality data collection will be critical for improvement. Additionally, a promising strategy would be to combine AI‐based rapid prescreening with more resource‐intensive quantum chemical methods for candidate prediction. (4) The probes we have discovered serve as valuable tools for imaging applications, particularly in two‐photon imaging modality that well‐suited to their emission profiles [53]. Nevertheless, the development of near‐infrared probes remains a priority, as it was not systematically explored in the present study. Future work should therefore focus on modification strategies such as extending π‐conjugation and implementing large‐scale screening.

In conclusion, we developed a hybrid AI‐driven framework for the discovery of target‐specific fluorescent probes, powered by our robust multi‐task predictive PROBY models. Using this approach, we successfully identified thousands of candidate probes for diverse targets. Furthermore, we validated candidate probes for TDP‐43, BCL‐2, and tau through in vitro imaging studies. Guided by PROBY, we developed a novel probe capable of in vivo two‐photon imaging and 3D transparent tissue imaging of tau aggregates based on the AI‐identified candidate, highlighting the practical utility of our strategy. This work establishes a generalizable and efficient pipeline for discovering the next‐generation of target‐specific fluorescence probes, with broad translational potential in biomedical research and clinical surgery navigation.

## 4 Experimental Methods

### 4.1 General Information

All chemicals and reagents for probe synthesis and characterization were obtained from commercial suppliers at analytical grade and used without further purification. Absorption spectra were acquired using a SHIMADZU UV‐2600 UV‐VIS spectrophotometer and fluorescence measurements were performed on a SHIMADZU RF‐5301 PC spectrophotometer. All the photophysical characterization experiments were carried out at room temperature. The human brain sections were obtained from the Chinese Brain Bank Center (CBBC, http://www.cbbcnet.cn) and were handled in accordance with the ethical guidelines of the South‐Central Minzu University's ethics and technology safety committee (approved protocol: 2024‐scuec‐046). All animal experiments strictly followed the guidelines approved by the ethics committee of the Children's Hospital of Chongqing Medical University (approved protocol: CHCMU‐IACUC20250429005, IACUC‐CQMU‐2025‐ 0517).

### 4.2 Data Collection

Fluorescence data were collected from multiple public sources to construct a comprehensive dataset for model training. Specifically, 1,500,413 molecule entries from sources of NMMLSC [54], PCMD [55], NCATS [56], SRMLSC [57], Tox21 [58], and BCCG [59] were downloaded from PubChem [26]; 20,236 fluorophore data reported by Joung et al. [27]. and [55],169 fluorophore data reported by Zhu et al. [25]. were obtained from the corresponding literature (Sci. Data and FluoDB, respectively); and 1,250 manually curated fluorescence dyes and probes (referred to as FL Data) were manually compiled from literature via PubMed [26]. For PubChem datasets, the tested data contains both fluorescent and non‐fluorescent molecules with fluorescence activity scores. To align the data, we set the scores of 20 as the threshold for categorizing fluorescent and non‐fluorescent. For other datasets, each molecule was annotated with detailed photophysical properties, including absorption and emission wavelengths, molar absorptivity, full width at half maximum (FWHM), lifetime and photoluminescence quantum yield across various solvents.

To eliminate invalid and redundant data, molecular structures were first encoded as SMILES (Simplified Molecular Input Line Entry System) strings [28], and invalid SMILES were removed. Duplicate entries with identical SMILES, test conditions (absorption and emission wavelengths), and fluorescence categories were then merged, resulting in a curated dataset comprising 1,169,719 unique entries. This integrated dataset was designated as FLP.

### 4.3 Data Preprocessing

To ensure consistency across datasets, we performed data preprocessing prior to model training.

For PROBY‐L1, SMILES entries with conflicting fluorescence category annotations were removed, resulting in a final dataset of 983,790 molecules for PROBY‐L1 model development.

For PROBY‐L2, only molecules with photophysical property data were included, yielding an initial dataset of 74,442 entries. We applied the same preprocessing protocol as previously published25. Briefly, entries lacking solvent information or using gaseous solvents were excluded. General filters were applied to retain data with quantum yield ≤ 1, absorption/emission wavelengths (*λ*_abs_/*λ*_em_) between 200–1500 nm, and molar absorptivity (*ε*_max_) ≤ 107. Parameter‐specific thresholds were also applied to remove inconsistent measurements: a 5 nm tolerance for *λ*_abs_/*λ*_em_, 0.1 for quantum yield, and 0.02 for log10 *ε*_max_. Outliers were excluded, and replicate entries were averaged. After preprocessing, the final dataset sizes for developing PROBY‐L2 were as follows: 31,358 for absorption wavelength, 25,575 for emission wavelength, 3,487 for absorption FWHM, 6,598 for emission FWHM, 26,274 for molar absorptivity, 19,582 for quantum yield, and 6,360 for fluorescence lifetime.

### 4.4 Model Development

Both models were developed by using Chemprop, a graph neural network framework for molecular property prediction^60^. Following data preprocessing, molecular graphs were generated from SMILES strings using RDKit. Atom‐level (atomic number, mass, valence, charge, chirality, hybridization) and bond‐level (aromaticity, conjugation, stereochemistry) features were encoded into feature vectors. The model utilized a bond‐centric message‐passing framework where each bond's features were updated by summing the features of neighboring bonds and concatenating them with the current bond's own features. These were then passed through neural network layers with nonlinear activation functions. After multiple iterations, atom features were pooled to produce molecular representations. These embeddings were then processed by a feed‐forward network for final property prediction.

PROBY‐L1 is a binary classification model for differentiating fluorescent and non‐fluorescent molecules.

The model takes two inputs: (i) the SMILES string representing the molecular structure, and (ii) the designated wavelength pair consisting of absorption (300–900 nm) and emission (400–1200 nm) wavelengths. The model output is an activity score between 0 (non‐fluorescent) and 1 (fluorescent), indicating fluorescence potential under the specified conditions.

PROBY‐L2 is a suite of regression models for predicting key photophysical properties across various solvent environments (absorption wavelength, emission wavelength, absorption FWHM, emission FWHM, molar absorptivity, photoluminescent quantum yield, and fluorescence lifetime). The model takes two inputs: (i) the SMILES string representing the molecular structure, and (ii) the SMILES string of designated solvents. Each model outputs a predicted value corresponding to one of the seven properties.

Both models were developed using a 7:1:2 split for training, validation, and test sets. Binary cross‐ entropy was used as the loss function for classification tasks, and mean squared error was employed for regression. The final configuration was set with a message‐passing depth of 3, a solvent‐specific depth of 3, a hidden size of 300, a solvent‐specific hidden size of 300. PROBY‐L1 was trained for 30 epochs with an initial learning rate of 0.0001, while PROBY‐L2 was trained for 200 epochs with an initial learning rate of 0.001.

Model performance was evaluated on held‐out test sets. For PROBY‐L1, classification performance was assessed using the area under the receiver operating characteristic curve (AUC). For PROBY‐L2, regression performance was evaluated using the coefficient of determination (*R^2^*) and mean absolute error (MAE). All performance metrics were calculated using scikit‐learn, and receiver operating characteristic (ROC) curves were generated by comparing predicted scores with experimentally measured fluorescence labels.

### 4.5 Two‐Step Deep Learning Pipeline for Candidate Probe Identification

We developed a two‐step deep learning pipeline to enable high‐throughput identification of candidate fluorescent probes.

In the first step, a library of molecules represented as SMILES strings was input into PROBY‐L1, along with designated excitation/emission wavelength pairs. To increase computational efficiency, fluorescence activity scores for molecules already present in the PROBY‐L1 training dataset were retrieved directly. For molecules not previously seen by the model, PROBY‐L1 generated predicted activity scores. Molecules with predicted scores below a predefined fluorescence threshold were excluded, while those exceeding the threshold were advanced to the second step for further analysis.

In the second step, the filtered fluorescent candidates were input into PROBY‐L2, along with the corresponding solvent condition (represented as SMILES). For molecules already present in the PROBY‐L2 dataset, the photophysical properties were retrieved. For unseen molecules, the model predicted 7 photophysical parameters.

This two‐step strategy enabled both rapid pre‐screening and refined regression‐based prioritization, facilitating the identification of candidates with desirable photophysical properties.

### 4.6 Candidate Probe Screening

In this study, the two‐step deep learning pipeline was applied to a library from TargetMol comprising 26,416 small molecules, including FDA‐approved drugs, clinically evaluated compounds, and preclinical candidates. Each molecule was annotated with validated target‐binding profiles. Application of our pipeline identified thousands of small molecules with both fluorescence potential and specific target engagement, offering a valuable resource for probe development.

To visualize molecular diversity, ECFP4 fingerprints (radius 2, 2048 bits) were generated using RDKit from canonical SMILES. Murcko scaffolds were extracted to define structural cores. t‐SNE (perplexity = 30, random state = 42) was applied to project the fingerprints into 2D space. The 20 most frequent scaffolds were visualized by converting their SMILES into molecular structures using RDKit and plotted using Matplotlib.

### 4.7 Protein Aggregates Preparation

The human recombinant tau protein (1 mg, QYAOBIO) was dissolved in PBS containing 110 µM heparin sodium to a final concentration of 55 µm. The solution was shake vigorously (100 rpm, Crystal IS‐RDV1) at 37°C for 72 h. Similarly, the human recombinant TDP‐43 protein (0.9 mg, Sangon Biotech) was dissolved in 1 mL Tris buffer (50 mm Tris, 300 mm NaCl, 10% Glycerol, pH 8.0) and was shake vigorously (200 rpm, Crystal IS‐RDV1) at 37°C for 48 h. Aggregates formation was monitored and verified using ThT fluorescence assay. The resulting aggregates was divided into aliquots and stored at −20°C before use.

### 4.8 Photophysical Property Characterization

For absorption and emission spectra measurements, the molecules were diluted to 10 µm in various solvents, including EtOH, DMSO, MeCN, H_2_O, and MeOH. Prior to analysis, the prepared samples were equilibrated at 25°C for 5 min to ensure consistent conditions. Subsequently, their absorption and emission spectra were measured. The absolute fluorescence quantum yield of these probes was measured using the FLS1000 Photoluminescence Spectrometer (Edinburgh Instruments Ltd.).

To evaluate the fluorescence turn‐on responses to targets, protein (2 µm) were incubated with probe solutions (2 µm, PBS containing 10% DMSO) for 60 min at room temperature. For probe B3, specific experiments involved co‐incubation of 5 µm B3 with 10 µm TDP‐43 aggregates for 60 min. Fluorescence was subsequently recorded at specified excitation wavelengths (PE859, *λ*_ex_ = 340 nm, *λ*_em_ = 360–900 nm; 859‐1, *λ*_ex_ = 490 nm, *λ*_em_ = 500–900 nm; 859‐2, *λ*_ex_ = 560 nm, *λ*_em_ = 590–800 nm; obatoclax, *λ*_ex_ = 480 nm, *λ*_em_ = 520–900 nm; B3, *λ*_ex_ = 370 nm, *λ*_em_ = 380–600 nm).

For selectivity evaluation, the probes were diluted to a final concentration of 1 µm and treated with 2 µm of various analytes. After 60 min of incubation at room temperature, fluorescence intensity was recorded at the specific excitation and emission wavelengths for each probe. The maximum emission wavelength intensity was plotted against the respective analytes.

For photostability evaluation, PE859, 859‐1, or 859‐1 were diluted to 10 µm in PBS containing 10% DMSO. The solutions were loaded into a fluorescence spectrometer and the fluorescent intensity at maximum emission wavelength was recorded under continuous excitation for 180 min.

### 4.9 Dissociation Constant Measurement

To 1 µm protein solution in PBS containing 10% DMSO, different concentrations of probe was added (final concentration 0, 1, 2, 5, 8, 10, 20, 50, 80, 100, 200, 500, 800, and 1000 nm). The mixture was dispensed into 96‐well plates and incubated at 37°C for 1 h. The fluorescence intensity of the samples was recorded at maximum emission wavelength of probe by using a microplate reader (PerkinElmer, VICTOR Nivo^TM^). Dose‐ response curves were generated by plotting fluorescence intensity against probe concentration, and equilibrium dissociation constants were calculated via nonlinear regression analysis in GraphPad Prism.

### 4.10 The Förster‐Hoffmann Equations

The relationship between the fluorescence intensity of 859‐1/859‐2 and the viscosity can be determined by the Förster –Hoffmann equation as following:

(1) logI=C+xlogη

where *η* represents the viscosity, *I* represents the fluorescence intensity of the probes, and *C* is a constant, where x represents the sensitivity of the fluorescent probe toward the viscosity.

### 4.11 Virtual Screening for TDP‐43 Ligands

A structure‐based virtual screening strategy was conducted using SchrÖdinger suite. The X‐ray structure of human TDP‐43 RRM1 domain with D169G mutation (PDB ID: 4Y00) was used as the receptor model, which was then prepared by removing the water, adding hydrogen atoms to the protein, and assigning OPLS4 force field. After protein preparation, the binding site of the protein was identified using SiteMap, which included the residue Gly169 as a key component. A chemical library comprising approximately 1,500,000 compounds (ChemDiv, https://www.chemdiv.com/) was subjected to primary screening via semi‐flexible docking with Glide SP. The top 10% compounds were retained for Prime MMGBSA rescoring. The top‐ranked 14066 molecules were filtered by following rules based on the following criteria: 1) Interacts with Leu120 at the base of the predicted binding site; 2) the score with Tyr123< −1.2 kcal/mol and 3) Glide score < −3.6, ∆GMMGBSA < −30 kcal/mol. This yielded 5445 compounds that were further prioritized for blood‐brain barrier (BBB) permeability using the following ADME filters: CNS > 0, MW < 450, PSA< 90, 2< logP < 5). The remaining 485 compounds were clustered using the Average‐Linkage algorithm, resulting in a total of 266 molecules being retained. Subsequently, these 266 compounds were docked into the active site of the receptor protein using the Induced Fit Docking protocol. Finally, 30 compounds were ultimately selected based on their IFD score and visual inspection.

### 4.12 Molecular Docking

Docking was performed using Schrodinger software and Cryo‐EM structure of tau (PDB ID: 5O3L). The ligands 859‐1 and 859‐2 were optimized using LigPrep module. All the other parameters involved in remained default. Docking was performed using Glide SP and the top‐ranked poses were analyzed for binding energy and interaction details.

### 4.13 Density Functional Theory (DFT) Calculation

Density functional theory (DFT) and time‐dependent density functional theory (TD‐DFT) were per‐ formed using the Gaussian 16 C.01 software. All calculations were performed using the conductor‐like polarizable continuum model (C‐PCM) with the dielectric constant of DMSO. In addition, the optical dielectric constant of DMSO was used for the absorption energy calculation. The structures of PE859, 859‐1, and 859‐2 were optimized using the ωB97X‐D functional and 6–31+G* basis set. At the optimized geometries, TD‐DFT calculations were performed using the same functional and basis set to obtain vertical excitation energies. For each molecule, the lowest‐energy excited state with a significant oscillator strength (greater than 0.7) was chosen to perform emission wavelength calculations and geometry optimization on the chosen excited state. The orbital plots were generated using Gauss View graphical user interface. The HOMO‐LUMO energy gaps were calculated at the optimized excited state geometries.

### 4.14 Drug Screening with B3 as a Fluorescent Reporter

The drug A1‐A5 was stored as 10 mm stock solution in DMSO. For screening, a mixture of B3 (1 µm final concentration) and A1‐A5 (20 µm final concentration) was incubated with TDP‐43 aggregates (6 µm final concentration) in 384‐well microplates (50 µL/well, LABAELECT 11614) at room temperature for 1 h. Negative (B3 alone) and positive (B3 + TDP‐43 aggregates) controls were used. Fluorescence signals were recorded using a microplate reader (PerkinElmer VICTOR Nivo^TM^) (excitation: 405 nm, emission: 460 nm). Inhibition rates were calculated as: Inhibition rates (%)  =  (FIMean_Max_‐FIDrug)/FIMean_Max_ × 100.

### 4.15 Microscale Thermophoresis (MST) Assay

Microscale thermophoresis experiments were performed using a NanoTemper Monolith Instrument (NT.115). Purified TDP‐43 (Sangon Biotech, 0.9 mg/mL in Tris buffer) was labeled using the Monolith Protein Labeling Kit RED‐tris‐NTA (MO‐L018, Nanotemper, Germany) according to the manufacturer's instructions. For binding measurements, 50 nM labeled TDP‐43 was mixed with serially diluted concentrations of A5 in PBS‐T buffer. Samples were loaded into MST premium capillaries, and thermophoresis traces were recorded. Data analysis was performed by the MO Affinity Analysis software (Nanotemper), and the data was fit using the specific binding with Hill model in GraphPad 8.3.0.

### 4.16 Pathological Staining and Imaging

Human brain tissues of AD or ALS patients and human neuroblastoma tissues were immersed in xylene three times for 5 min to remove paraffin, followed by two 10 min washes with a series of diluted alcohols (100, 90%, 70%, and 50% ethanol in dd water, v/v). Following PBS washing, heat‐mediated antigen retrieval was per‐ formed in 10 mm citrate buffer (pH 9.0) using a temperature‐gradient protocol: samples were gradually heated to boiling point through sequential low‐to‐high intensity thermal exposure (total 15 min duration), followed by natural cooling to ambient temperature. Sections were then blocked with 5% goat serum in PBS for 1 h at room temperature, followed by incubation with primary anti‐tau antibody (1:400, Proteintech, Cat 66499‐1‐lg), anti‐ TDP‐43 antibody (1:500, Proteintech, Cat 10782‐2‐AP), or anti‐BCL‐2 antibody (1:500, Affinity, BF9103‐50 µL) for 24 h at 4°C. After washing with PBS‐T, the sections were labeled with secondary antibodies. To avoid the fluorescence overlap between antibody staining and probe labeling, specific secondary antibodies were selected. Goat Anti‐Mouse IgG (H+L) Fluor594‐conjugate (1:500, Proteintech, Cat RGAM004) was selected for confirming PE859 staining. Goat Anti‐Mouse IgG(H+L) Dylight405 (1:500, EarthOx, Cat E032110‐01) was selected for confirming 859‐1 staining. Goat Anti‐Mouse IgG(H+L) Dylight405 (1:500, EarthOx, Cat E032110‐01) was selected for confirming 859‐2 staining. AF647‐labeled Goat Anti‐Rabbit IgG (H+L) (1:500, Beyotime, Cat A0468) was selected for confirming B3 staining. Goat Anti‐Mouse IgG(H+L) Dylight405 (1:500, EarthOx, E032110‐01) was selected for confirming obatoclax staining. Subsequently, the sections were stained with PE859, 859‐1, 859‐2, or B3 for 10 min, sections were subjected to three sequential 5‐min washes in PBS containing 40% ethanol (v/v), then mounted with anti‐fade medium (Beyotime Biotechnology, China).

The fluorescence images of tissues were acquired by a scanning microscope (KF‐FL‐020, KFBIO). The selection of fluorescence channels were shown below: PE859, *λ*_ex_ = 375 ± 28 nm, *λ*_em_ = 431 ± 28 nm; 859‐1, *λ*_ex_ = 490 ± 20 nm, *λ*_em_ = 525 ± 20 nm; 859‐2, *λ*_ex_ = 546 ± 10 nm, *λ*_em_ = 572 ± 23 nm; obatoclax, *λ*_ex_ = 490 ± 20 nm, *λ*_em_ = 525 ± 20 nm; B3, *λ*_ex_ = 375 ± 28 nm, *λ*_em_ = 431 ± 28 nm; tau antibody, *λ*_ex_ = 546 ± 10 nm, *λ*_em_ = 572 ± 23 nm; TDP‐43 antibody, *λ*_ex_ = 592 ± 21 nm, *λ*_em_ = 630 ± 30 nm; BCL‐2 antibody, *λ*_ex_ = 375 ± 28 nm, *λ*_em_ = 431 ± 28 nm.

### 4.17 Cell Imaging

Cells of the logarithmic growth phase were taken and transferred them to centrifuge tubes (5 × 10^4^ cells/mL). After centrifugation, the culture medium was discarded. Cells were washed once with PBS, centrifuged, and the supernatant aspirated. One milliliter of ice methanol was added, followed by gentle resuspension and fixation for 30 min. After centrifugation, the supernatant was discarded, and cells were washed twice with PBS. Subsequently, 500 µL of 5% BSA blocking solution was added, and cells were gently resuspended before transfer to a 35‐mm confocal dish for sealing and incubation at room temperature for 1 h. The cell suspension was then added into a centrifuge tube, centrifuged to remove blocking solution, and resuspended in 500 µL of primary antibody solution (Proteintech, 12789‐1‐AP, 1:500). Cells were transferred to a confocal dish and incubated overnight at 4°C. After centrifugation to remove primary antibody, cells were washed twice with PBS. Next, 500 µL of secondary antibody (Beyotime, A0468, 1:500) was added, followed by incubation at room temperature for 1 h. Following centrifugation, the cells were washed twice with PBS and stained with 500 µL of DAPI solution (Beyotime, C1006) for 10 min at room temperature. After DAPI removal and two additional PBS washes, cells were treated with 5 µm obatoclax (EtOH/PBS = 2:3, v/v) for 10 min at room temperature. After centrifugation, cells were washed twice with PBS, resuspended in 1 mL PBS, transferred to a confocal dish, and imaged with a laser confocal microscope.

### 4.18 Tumor Model Imaging Study

SU‐DHL‐6 cells were suspended in 100 µL of 50% Matrigel / PBS mixture. Hun dred microliters of SU‐DHL‐6 cell suspensions, corresponding to 1.8 × 10^6^ cells per mouse, were subcutaneously injected into the left flank region of NSFG mice (female, aged 8–10 weeks). The mice were monitored for tumor formation. After 10–14 days of SU‐DHL‐6 cells seeding, obatoclax was dissolved in a PBS solution (containing 9.6% polyethylene glycol 300, 0.4% polysorbate 20 and 5% dextrose, 1 mg/mL) and injected into the tail vein (10 mg/kg) using a 1 mL insulin syringe. Mice were sacrificed after 4 h and tissues (heart, liver, spleen, lung, kidney, and tumor) were obtained. The fluorescence signals in tissues were recorded using the AmiHTX imaging system.

### 4.19 Blood‐Brain Barrier (BBB) Permeability Study

859‐2 (4 mg/kg, dissolved in PBS containing 15% DMSO and 15% cremophore) was intravenously administrated to 8‐week old Balb/c mice through the tail (20–22 g, female). Mice were euthanized to collect blood and whole brain at 0, 15, 30, 45, and 60 min after probe administration. Blood plasma was collected into a tube containing sodium heparin (20 mg/mL, 20 µL). Then, 50 µL of plasma was taken, mixed with 950 µL of acetonitrile (containing 1% formic acid, v/v), and vortexed for 3 min. The mixture was then placed in a −20°C refrigerator for 20 min to allow protein precipitation. Following this, it was centrifuged at 4°C for 20 min at 15,000 rpm. The resulting supernatant was used for subsequent LC‐MS analysis.

Brains were homogenized in 1 mL acetonitrile (containing 1% formic acid, v/v). The brain homogenates were then vortexed for 2 min, and then placed in a ‐20°C refrigerator for 20 min to allow protein precipitation. Following this, it was centrifuged at 4°C for 20 min at 15,000 rpm. The resulting supernatant was used for subsequent LC‐MS analysis.

Brain homogenate and plasma samples from untreated mice were prepared as above. 859‐2 was added to aliquots of these samples at concentrations ranging from concentration range ng/mL. Samples were mixed with acetonitrile and then placed in a ‐20°C refrigerator for 20 min to allow protein precipitation. Following this, it was centrifuged at 4°C for 20 min at 15,000 rpm. The resulting supernatants were analyzed by LC‐MS analysis to give the standard curves.

### 4.20 Two‐Photon In Vivo Imaging

Twenty‐four hours before imaging, tau P301S (Line PS19) mice were injected with FSB (reported tau‐targeted dye, 7.5 mg/kg, i.p, dissolved in 10% DMSO with PBS). On the imaging day, mice were were anesthetized and a skull optical clearing window was established using an in vivo skull optical clearing kit (JA6011, Jarvis Biological Pharmaceutical Co., Ltd). The steps were conducted as follows: S1 (saturated solution of urea dissolved in ethanol) was administered to the revealed skull for 10 min, followed by applying S2 (sodium dodecylbenzenesulfonate solution) liberally for 5 min. Before injecting 859‐2, the FSB signal at 800 and 1000 nm channels were recorded by a two‐photon laser scanning microscopy (A1R MP, Nikon). Subsequently, 859‐2 (4 mg/kg, dissolved in PBS containing 15% DMSO and 15% cremophore) was injected through the tail vein, and the mice brains were observed at 30 min postinjection. All images were collected using a 25 × water immersion objective (NA = 1.1, WD = 2 mm) with 1024 × 1024 pixel (z‐step = 2 µm, speed = 0.125 frame/s). Imaging channel for FSB was *λ*_ex_ = 800 nm, *λ*_em_ = 500–550 nm and channel for 859‐2 was *λ*_ex_ = 1000 nm, *λ*_em_ = 600–660 nm.

### 4.21 fMOST Brain Imaging

After two‐photon imaging, mice were anesthetized by isoflurane and were transcardially perfused with ice‐cold 1×PBS followed with ice‐cold 4% PFA solution. Brains were fixed in 4% PFA at 4°C for 24 h. After fixation, brains were rinsed with PBS overnight and subsequently dehydrated in a graded ethanol solution of 50%, 75%, 95% and 100% GMA (Ted Pella Inc., Redding, CA, USA) (each gradient for 2 h), and then soaked in 100% GMA for 3 days, and cured for 24 h in an oven at 48°C. The brain was installed in the fMOST system (BioMapping5000, Wuhan OE‐Bio Co., Ltd), which uses 40× water immersion eyepiece (0.8 NA, LUMPLFLN, Olympus) and TDI‐CCD (4K, DALSA) for signal detection. Brains were cut into ultrathin slices (thickness = 2 µm) continuously, and at the same time, the slices were imaged in turn.

### 4.22 3D Transparent Brain Imaging

After two‐photon imaging, mice were anesthetized by isoflurane and were transcardially perfused with ice‐cold 1×PBS followed with ice‐cold 4% PFA solution. Brain was incubated in 4% PFA at 4°C with shaking overnight. The samples were then washed with 1×PBS for at least 2 h twice at room temperature. The clearing of the fixed samples was conducted with Nuohai enhanced tissue clearing kit (NH‐CR‐230701, Nuohai Life Science (Shanghai) Co., Ltd). Briefly, samples were immersed individually in 20 mL of solution A for 7–8 days (refreshing the solution every two days) with gentle shaking at 37°C. The sample was washed in 1×PBS for 6 h with gentle shaking at room temperature and the PBS refreshed every 2 h. Finally, the samples were RI matched for 3D imaging. 3D fluorescence imaging of the cleared tissues was conducted with a Nuohai LS 18 tiling light sheet microscope (Nuohai Life Science Co., Ltd). A 4‐tile tiling light sheet was used to illuminate the samples, and a 1 × 0.25 NA objective (Olympus MVPLAPO) was used to collect the fluorescence. The magnification of the microscope was set at 4×, and the spatial resolution was roughly 3.3 × 3.3 × 7 µm^3^ at the selected imaging conditions. The collected images were processed with the LS 18 image combine software (Nuohai Life Science Co., Ltd) and rendered using Amira (Thermo Fisher Scientific, USA). Imaging channel for FSB was *λ*_ex_ = 405 nm, *λ*_em_ = 525 ± 25 nm, and channel for 859‐2 was *λ*_ex_ = 637 nm, *λ*_em_ = 698 ± 70 nm.

### 4.23 Statistical Analysis

All experiments were conducted over 3–5 independent experiments, and error bars represent the S.E.M across these runs. For paired model comparisons, we used a two‐sided Wilcoxon signed‐rank test to deter‐ mine statistical significance. Additionally, we employed the Pearson correlation coefficient and its associated two‐sided p‐value (computed using scikit‐learn's default method) to assess correlations between continuous variables. All statistical analyses and data visualizations were performed using GraphPad Prism 8.3.0 and Origin2021. Statistical analysis was performed using GraphPad Prism 8.3.0, with significance determined by one‐way ANOVA followed by Tukey's or Dunnett's post hoc tests. A p‐value below 0.05 was considered statistically significant. Colocalization analysis was performed using the Pearson's correlation coefficient (PCC) calculated via ImageJ software.

### 4.24 Computing Hardware and Software

We implemented all experiments using Python (version 3.8.13) with PyTorch (2.0.0) as the primary deep learning framework. The model training was conducted on an NVIDIA RTX A6000 GPU. The model was based on implementations from chemprop (v1.6.1). Data management and analysis were performed using NumPy (v1.23.4) and Pandas (v2.1.3). Visualization of the results and model outputs was carried out using Matplotlib (v3.8.3) and Seaborn (v0.13.2).

## Conflicts of Interest

The authors declare no conflicts of interest.

## Code Availability

The code is publicly available at https://github.com/18lyb12/proby, which includes installation instructions, model weights, the dataset, and example data.

## Supporting information




**Supporting File**: advs73409‐sup‐0001‐SuppMat.pdf.

## Data Availability

FluoDB is available at https://doi.org/10.6084/m9.figshare.26317933. NMMLSC, PCMD, NCATS, SRMLSC, Tox21, and BCCG dataset sources downloaded from PubChem is available at https://pubchem.ncbi.nlm.nih.gov/. Datasets from Sci. Data (DB for Chromophores) is available at https://figshare.com/articles/dataset/DB_for_chromophore/12045567/2?file=23637518.
