# Supplementary material for: AI‐Driven Acceleration of Fluorescence Probe Discovery
Source: Adv Sci (Weinh). 2025 Dec 24;13(10):e15604. doi: 10.1002/advs.202515604 (PMC12915096; doi:10.1002/advs.202515604)
Supplement: Supplementary file 1 — Supporting File: advs73409‐sup‐0001‐SuppMat.pdf. [file ADVS-13-e15604-s001.pdf]

Supplementary Information

Materials and instruments for synthesis and structure characterization

All chemicals and reagents for probe synthesis were obtained from commercial suppliers at analytical grade and used without further purification.e region of interest. ^{1}H and ^{13}C NMR spectra were recorded on a Bruker spectrometer (400 MHz). All NMR spectra were calibrated using the residual solvent (DMSO) as internal reference (^{1}H NMR = 2.5, ^{13}C NMR = 39.5). All chemical shifts were reported in parts per million (ppm) and coupling constants (J) in Hz. High resolution mass spectra (HRMS) were performed in OPEN ACCESS R&D Laboratories.

Probe synthetic methods

Synthesis of 2,2-difluoro-4,6-dimethyl-1-oxa-3-oxonia-2-boranuidacyclohexa-3,5-diene (2)

To a solution of pentane-2,4-dione (1 g, 9.99 mmol, 1.03 mL, 1 eq) in DCM (10 mL) was added K_2CO_3 (1.38 g, 9.99 mmol, 1 eq) and $BF_3\cdot$ E_2O (2.95 g, 9.99 mmol, 2.56 mL, 48% purity, 1 eq). The mixture was stirred at 20 °C for 2 h under N_2. TLC indicated pentane-2,4-dione was consumed completely and one new spot formed. The reaction mixture was quenched by H_2O 20 mL at 0 °C, and stirred at 20 °C for 0.5 h, The mixture was extracted with EtOAc 30 mL (10 mL × 3). The combined organic layers were washed with brine 10 mL, dried over Na_2SO_4, filtered and concentrated under reduced pressure to give a residue. The residue was purified by column chromatography (SiO_2, Petroleum ether : Ethyl acetate = 50 : 1 to 0 : 1, *v/v*). Compound 2 was obtained as a yellow solid, (1.3 g, 87.99%). ^{1}H NMR (400 MHz, DMSO-d_6) δ 6.38 (s, 1 H), 2.33 (s, 6 H).

Synthesis of 2-methoxy-4-(2-pyridylmethoxy) benzaldehyde (3a):

To a solution of 4-hydroxy-2-methoxy-benzaldehyde (200 mg, 1.31 mmol, 1 eq) in DMF (10 mL) was added K_2CO_3 (181.67 mg, 1.31 mmol, 1 eq) and 2-(chloromethyl) pyridine hydrochloride (167.69 mg, 1.02 mmol, 0.78 eq). The mixture was stirred at 50 °C for 8 h. TLC indicated 4-hydroxy-2-methoxy-benzaldehyde was consumed completely and three new spots formed. The reaction mixture was diluted with H_2O 20 mL and extracted with EtOAc 45 mL (15 mL × 3). The combined organic layers were washed with brine 20 mL (10 mL × 2), dried over Na_2SO_4, filtered and concentrated under reduced pressure to give a residue. The residue was purified by column chromatography (SiO_2, Petroleum ether : Ethyl acetate= 10 : 1 to 2 : 1, *v/v*). Compound 4a was obtained as a gray solid, (250 mg, 78.18% yield). ^{1}H NMR (400 MHz, DMSO-d_6) δ 10.18 (s, 1H), 8.60 (dd, J = 4.9, 0.9 Hz, 1H), 7.86 (td, J = 7.7, 1.8 Hz, 1H), 7.67 (d, J = 8.7 Hz, 1H), 7.55 (d, J = 7.8 Hz, 1H), 7.36-7.39 (m, 1H), 6.84 (d, J = 2.3 Hz, 1H), 6.74 (dd, J = 8.7, 1.4 Hz, 1H), 5.31 (s, 2H), 3.90 (s, 3H).

Synthesis of 6-[(*E*)-2-[2,2-difluoro-6-[(*E*)-2-[2-methoxy-4-(2-pyridylmethoxy) phenyl] vinyl]-3-oxa-1-oxonia-2-boranuidacyclohexa-4,6-dien-4-yl] vinyl]-1*H*-indole (859-1).

To a solution of 2-methoxy-4-(2-pyridylmethoxy) benzaldehyde (3a) (100 mg, 411.09 μmol, 1 eq) and 1*H*-indole-6-carbaldehyde (59.67 mg, 411.09 μmol, 1 eq) in Tol. (5 mL) was added 2,2-difluoro-4,6-dimethyl-1-oxa-3-oxonia-2-boranuidacyclohexa-3,5-diene (60.81 mg, 411.09 μmol, 1 eq), tributyl borate (189.22 mg, 822.17 μmol, 221.83 μL, 2 eq) and n-BuNH2 (18.03 mg, 82.22 μmol, 0.2 eq). The mixture was stirred at 65 °C for 8 h. The reaction mixture was concentrated under reduced pressure to remove Tol (5 mL). The residue was purified by prep-HPLC (column: Phenomenex Luna C18 100 × 30mm, 5 μm; mobile phase: H_2O (0.1%

TFA)-ACN]; gradient:35%-65% B over 8.0 min). Compound 859 was obtained as a brown solid, (114 mg, 45.14% yield, TFA). ^{1}H NMR (400 MHz, DMSO-d_6) δ 11.59 (s, 1H), 8.59-8.61 (m, 1 H), 8.13 (dd, J = 15.6, 2.7 Hz, 2H), 7.80 – 7.91 (m, 3H), 7.64 (d, *J* = 8.4 Hz, 1H), 7.54-7.59 (m, 3H), 7.36-7.39 (m, 1H), 7.09 (dd, *J* = 28.4, 15.7 Hz, 2H), 6.83 (d, *J* = 2.3 Hz, 1H), 6.77 (dd, *J* = 8.7, 2.4 Hz, 1H), 6.52-6.54 (m, 2H), 5.31 (s, 2H), 3.94 (s, 3H). ^{13}C NMR (400 MHz, DMSO-d_6) δ 178.81, 178.66, 163.33, 160.82, 156.00, 149.21, 148.64, 140.57, 137.12, 135.97, 131.48, 131.09, 129.73, 127.32, 123.21, 122.02, 120.80, 119.44, 118.55, 118.19, 116.05, 115.30, 107.76, 102.27, 101.62, 99.37, 70.77, 56.08. HR-MS (*m/z*): $[M + H]^+$ calc'd. for $C_{28}H_{24}BF_2N_2O_4$: 501.1797, found 501.1783.

Synthesis of (*E*) - 4 - (2-(2,2-difluoro-6-methyl-2H-1λ^3,3,2λ^4-dioxaborinin-4-yl) vinyl) - *N*, *N* -dimethylaniline (3b):

To a solution of 2,2-difluoro-4,6-dimethyl-1-oxa-3-oxonia-2-boranuidacyclohexa-3,5-diene (500 mg, 3.38 mmol, 1 eq) and 4-(dimethylamino) benzaldehyde (504.31 mg, 3.38 mmol, 548.16 μL, 1 eq) in Toluene (10 mL) was added butan-1-amine (54.39 mg, 743.67 μmol, 73.50 μL, 0.22 eq) and tributyl borate (1.71 g, 7.44 mmol, 2.01 mL, 2.2 eq) at 0 °C. Then the mixture was warmed to 65 °C and stirred for 8 hours. The reaction mixture was diluted with H_2O 50 mL and extracted with ethyl acetate 90 mL (30 mL × 3). The combined organic layers were washed with brine 50 mL (25 mL × 2), dried over Na_2SO_4, filtered and concentrated under reduced pressure to give a residue. The residue was purified by column chromatography (SiO_2, Petroleum ether : Ethyl acetate = 5 : 1 to 0 : 1, *v/v*). Compound 3b (600 mg, 2.15 mmol, 63.60% yield) was obtained as a gray solid. ^{1}H NMR (400 MHz, DMSO-d_6) δ 7.82 (d, *J* = 14.6 Hz, 1H), 7.68 (d, *J* = 8.4 Hz, 2H), 6.70-6.79 (m, 4 H), 3.06 (s, 6H), 1.23 (s, 3H).

Synthesis of (*E*)-4-(2-(pyridin-2-yl)vinyl)benzaldehyde (4b):

To a solution of 2-methylpyridine (1 g, 10.74 mmol, 1.06 mL, 1 eq) in acetyl acetate (3.88 g, 38.03 mmol, 3.57 mL, 3.54 eq) and AcOH (2.25 g, 37.43 mmol, 2.14 mL, 3.49 eq) was added terephthalaldehyde (2.16 g, 16.11 mmol, 1.5 eq). The mixture was stirred at 135 °C for 12 h. The reaction mixture was diluted with H_2O 30 mL and adjust pH to 7 with sat. Na_2CO_3 extracted with ethyl acetate 15 mL (5 mL×3). The combined organic layers were washed with brine 50 mL (25 mL×2), dried over Na_2SO_4, filtered and concentrated under reduced pressure to give a residue. The residue was purified by column chromatography (SiO_2, Petroleum ether : Ethyl acetate = 10 : 1 to 2 : 1, *v/v*). Compound 4b (1.2 g, 5.73 mmol, 53.41% yield) was obtained as a white solid.

Synthesis of 4 - ((*E*)-2-(2,2-difluoro-6-((*E*)-4-((*E*)-2-(pyridin-2-yl) vinyl) styryl) - 2H - 1λ^3,3, 2λ^4-dioxaborinin-4-yl) vinyl) - *N*, *N* - dimethylaniline (859-2):

To a solution of 4-[(*E*)-2-(2-pyridyl) vinyl] benzaldehyde (300 mg, 1.43 mmol, 1 eq) and 4-[(*E*)-2-(2,2-difluoro-6-methyl-3-oxa-1-oxonia-2-boranuidacyclohexa-4,6-dien-4-yl) vinyl] – *N*, *N* - dimethyl-aniline (400.14 mg, 1.43 mmol, 1 eq) in Toluene. (20 mL) was added tributyl borate (659.95 mg, 2.87 mmol, 773.68 μL, 2 eq) and butylamine (23.07 mg, 315.42 μmol, 31.18 μL, 0.22 eq). The mixture was stirred at 65 °C for 8 hours. The reaction mixture was diluted with H_2O 20 mL and extracted with ethyl acetate 45 mL (15 mL × 3). The combined organic layers were washed with brine 20 mL (10 mL × 2), dried over Na_2SO_4, filtered and concentrated under reduced pressure to give a residue. The residue was purified by prep-HPLC (column: Phenomenex Luna C18 100 × 30 mm, 5 μm; mobile phase: [H_2O (0.1% TFA)-ACN]; gradient: 20%-55% B over 8 min). Compound 859-2 (40 mg, 64.84 μmol, 16.94% yield, 94.72% purity, TFA) was obtained as a black brown solid. ^{1}H NMR (400 MHz, DMSO-d_6) δ 8.63 – 8.68 (m, 1H), 7.94 – 8.05 (m, 2H), 7.88 (d, *J* = 7.8 Hz, 2H), 7.82 (d, *J* = 5.9 Hz, 1H), 7.74-7.79 (m, 6H), 7.48 (d, *J* = 16.2 Hz, 1H), 7.40-7.44 (m, 1H), 7.18

(d, J = 15.8 Hz, 1H), 6.88 (d, J = 15.2 Hz, 1H), 6.82 (d, J = 9.0 Hz, 2H), 6.44 (s, 1H), 3.10 (s, 6H). ^{13}C NMR (400 MHz, DMSO-d_6): δ 180.13, 175.54, 154.05, 153.89, 149.83, 142.96, 139.61, 139.11, 135.35, 133.65, 133.33, 130.07, 128.41, 123.73, 122.73, 122.07, 114.24, 112.64, 102.33. HR-MS (*m/z*): $[M + H]^+$ calc'd for $C_{28}H_{26}BF_2N_2O_2$ 471.2055, found.471.2065.

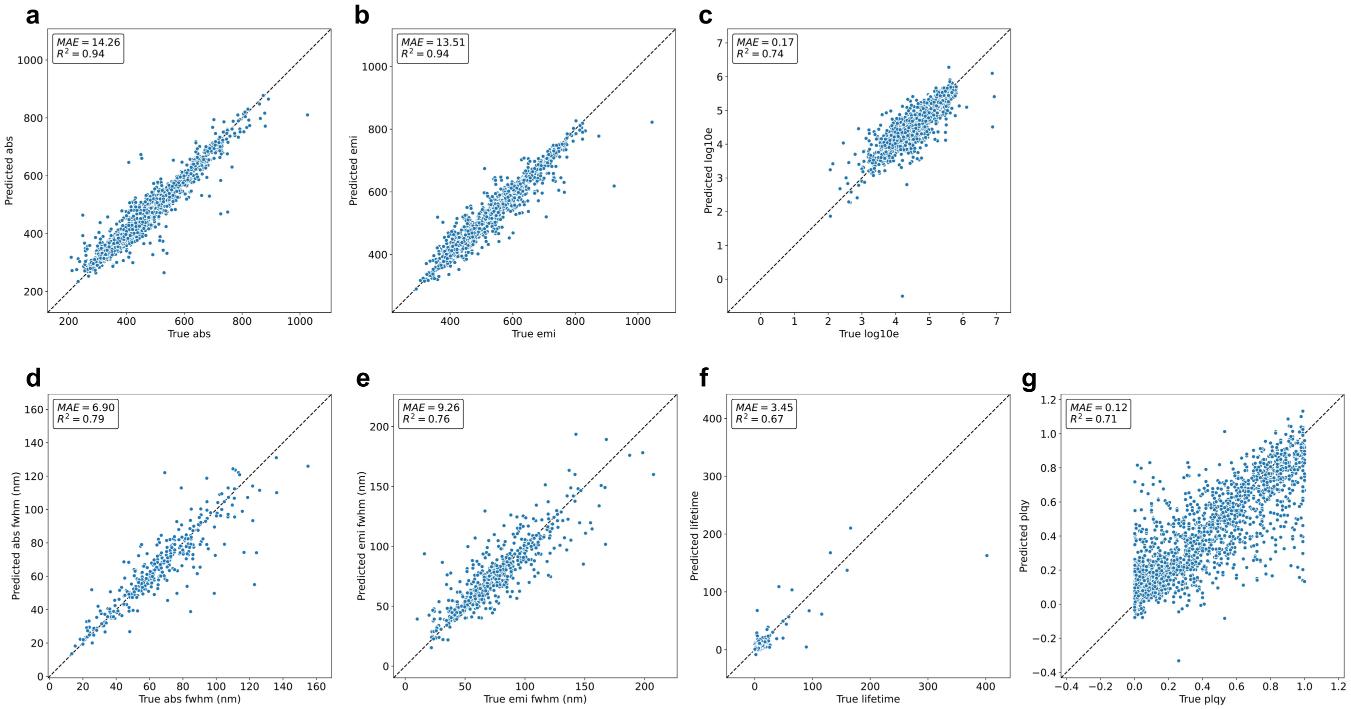

Extended Data Figure 1: **Parity plot of the optical properties predicted by PROBY-L2.** PROBY-L2 showed strong predictive performance on validation datasets for predicting the absorption wavelengths (n = 3,136 in (**a**)), emission wavelengths (n = 2,558 in (**b**)), molar absorptivity (n = 2,628 in (**c**)), absorption full width at half maxima (FWHM) (n = 349 in (**d**)), emission full width at half maxima (FWHM) (n = 660 in (**e**)), lifetime (n = 636 in (**f**)), and quantum yield (n = 1,958 in (**g**)).

a

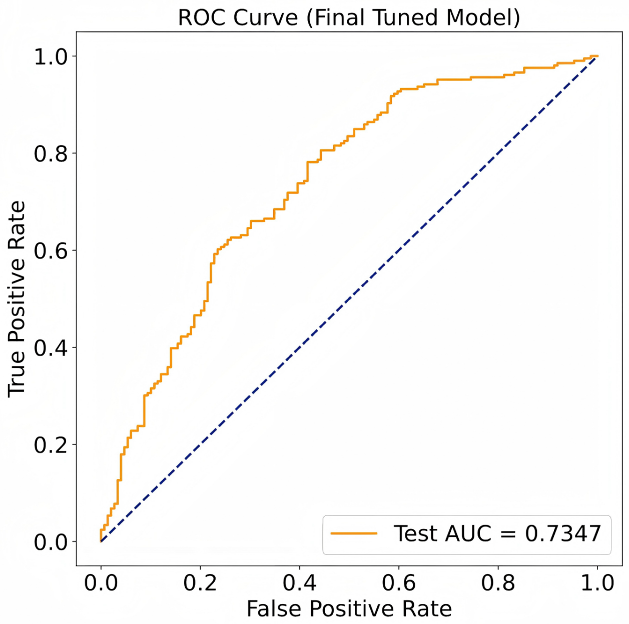

b

Compound ID	predict	test
T7003	0	0
T4077(PE859)	1	1
T6888	0	0
4852-0197	0	0
2079-0021	1	0
E201-0354 (B3)	1	1
E201-0140	1	0
K505-0424	0	0
T6275(Obatoclax)	0	1
T6S0052	1	0
T6778	0	0

Extended Data Figure 2: **Performance of the fluorogenic prediction model and experimental validation.** **(a)** Model performance on the fluorogenic prediction task, achieving an area under the curve (AUC) of 0.73 on the test dataset. **(b)** Comparison between model predictions and experimental results for 11 candidate molecules. The model correctly predicted the turn-on behavior for 7 out of 11 molecules.

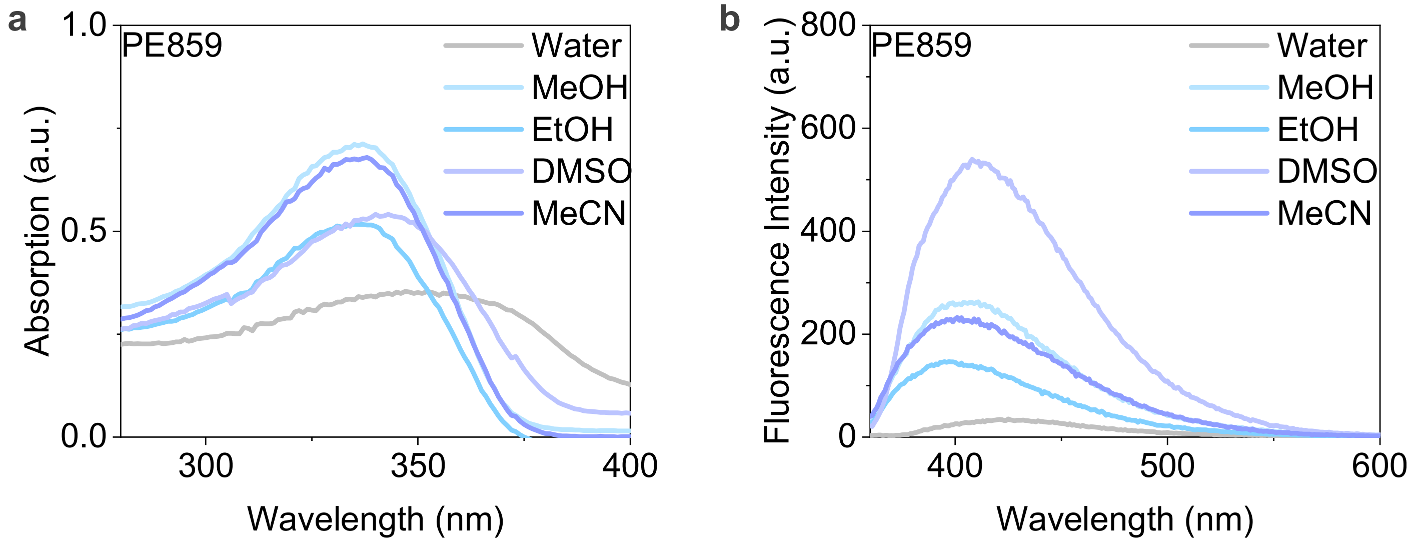

Extended Data Figure 3: **Photophysical properties of PE859 in various organic solvent.** PE859 was dissolved in water, methanol (MeOH), ethanol (EtOH), dimethyl sulfoxide (DMSO), and acetonitrile (MeCN) at a concentration of 10 μM, respectively. The absorption (**a**) and (**b**) emission spectra were measured. PE859 emitted weak signal in water, but the signal intensity is significantly enhanced in organic solvents with lower polarity. Additionally, the emission wavelength of PE859 displayed a blue shift in these less polar solvents. These results suggested that PE859 is highly sensitive to its surrounding environment.

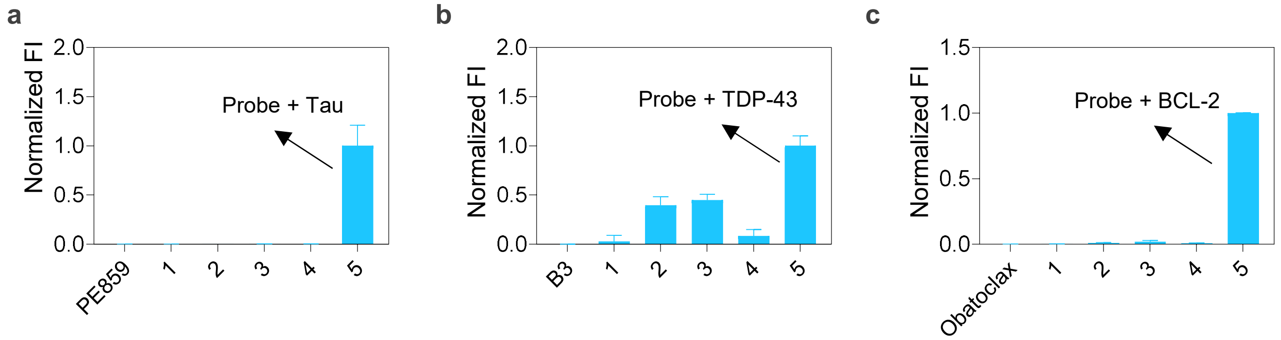

Extended Data Figure 4: **Selectivity of fluorescent probes PE859, B3, and Obatoclax.** Fluorescence response upon addition of various proteins and biomolecules (1. Trypsin, 2. HSA, 3. Lysozyme, 4. BSA, 5. Target (Tau aggregates, TDP-43 aggregates or BCL-2 protein). All data were normalized to the probe-only control, showing minimal response to non-target analytes. The data are the mean ± S.E.M., n = 3.

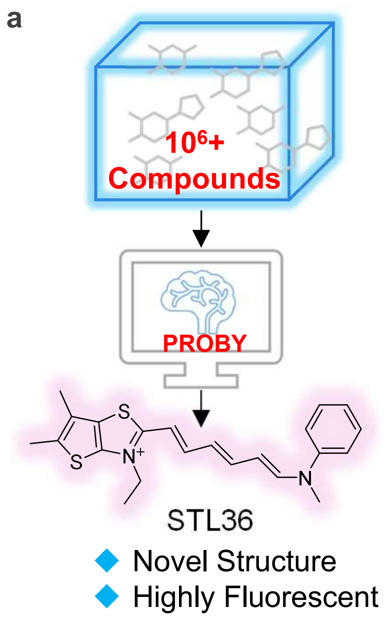

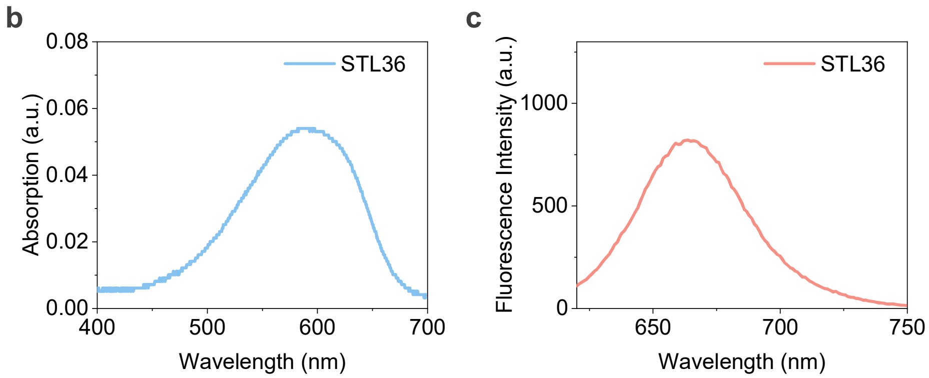

d

Compound	$\lambda_{Abs}{}^{a}$	$\lambda_{em}{}^{a}$	$\lambda_{Abs}{}^{b}$	$\lambda_{em}{}^{b}$	$\log_{10\varepsilon}{}^{a}$	$\log_{10\varepsilon}{}^{b}$
STL36	565	697	600	664	4.6078	4.6421

a：predicted data by PROBY; b：experimental data

Extended Data Figure 5: **Discovery and optical characterization of the novel fluorophore STL36 guided by the PROBY model. a.** Schematic of the virtual screening workflow that led to the identification of the previously unreported fluorophore STL36. **b, c.** Normalized experimental absorption (b) and fluorescence emission spectra (c) of STL36 (10 μM) in DMSO. **d.** Comparison between the predicted and experimental data, showing reasonable agreement and demonstrating the model's predictive capability.

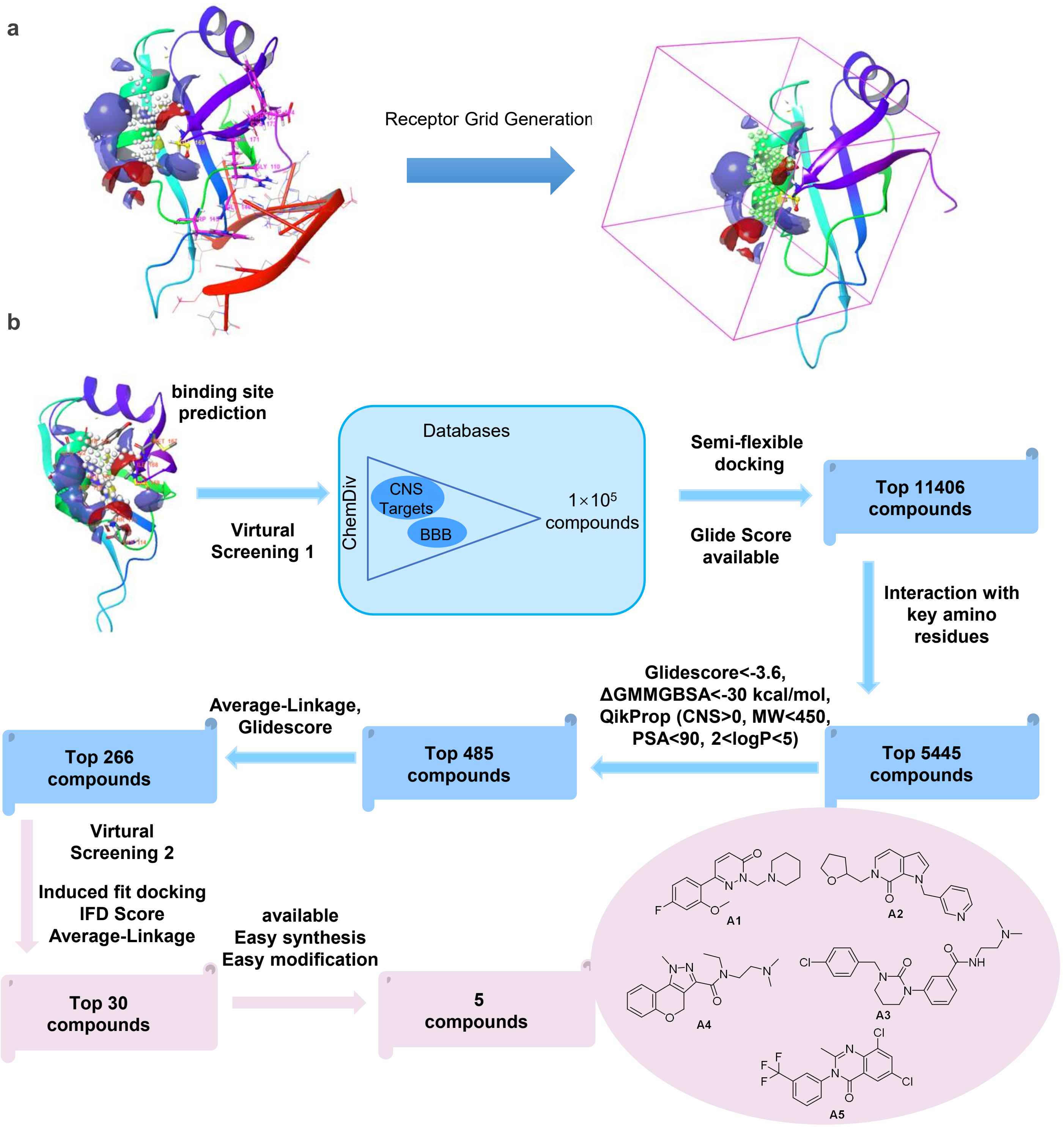

Extended Data Figure 6: **Workflow and results of computational docking experiments for screening new drugs targeting TDP-43 protein.** **a.** Predicted binding pocket of the TDP-43 protein (PDB ID: 4Y00) prior to screening. Left: Binding site identified by SiteMap. Right: Docking grid region (red box; 10 × 10 × 10 Å) used for subsequent molecular docking. **b.** Computational docking screening workflow. A compound library of 100,000 molecules from ChemDiv was docked into the predicted TDP-43 binding site. In the first round, semi-flexible docking using Glide SP identified 266 candidates based on Glide score, physicochemical properties, and clustering. In the second round, flexible docking and clustering yielded 21 molecular scaffolds. From these, 30 top candidates were selected based on predicted binding affinity, of which 5 representative molecules were selected based on availability, synthetic feasibility, and potential for further chemical modification.

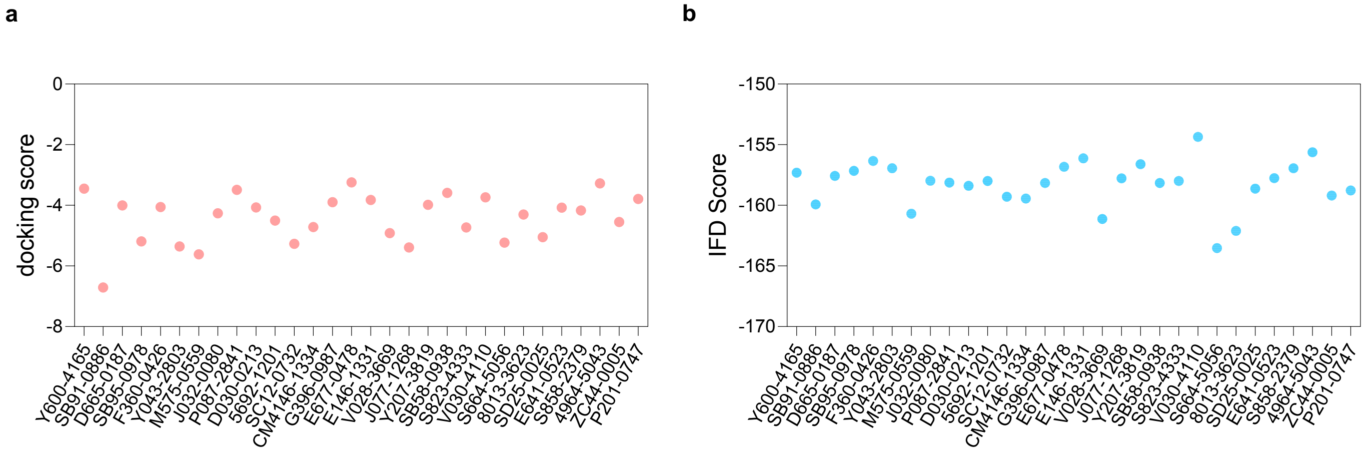

Extended Data Figure 7: **Computational docking results of top 30 compounds targeting TDP-43 protein.** After two rounds of virtual screening, the compounds with good docking scores (**a**) and IFD scores (**b**) were identified. Compound names were shown as Chemdiv ID.

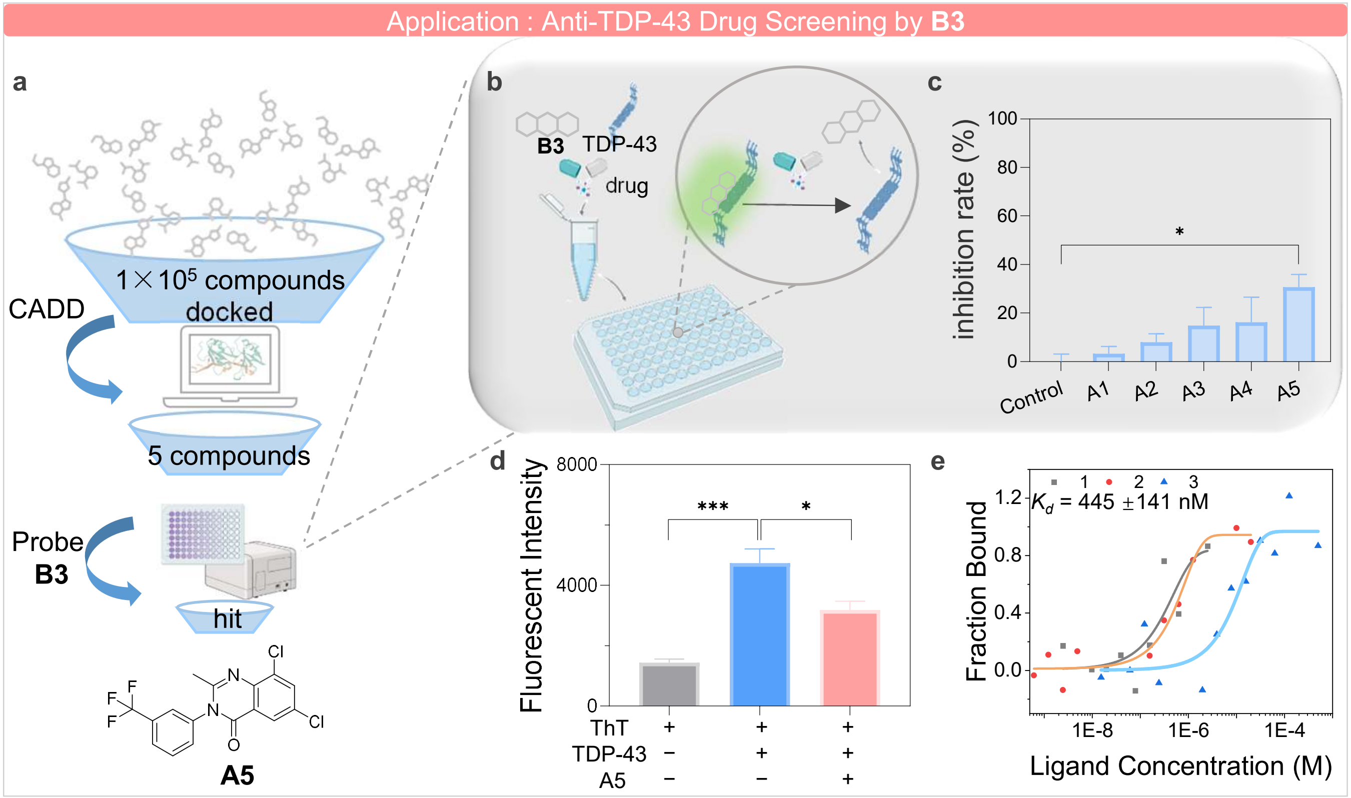

Extended Data Figure 8: **Drug screening applications of B3.** **a.** Computational-experimental pipeline for TDP-43 ligand discovery. Virtual screening of over 100,000 compounds against TDP-43 (PDB: 4Y00) yielded 5 candidate compounds, with probe B3 fluorescence assays confirming A5 as a novel ligand. Diagram of the TDP-43-targeted drug screening workflow by combining computational methods and probe B3. Over 100,000 compounds were first screened by Computer-Aided Drug Design (CADD) with TDP-43 proteins (PDB ID: 4Y00), resulting in 5 promising candidate ligands. Next, probe B3 was used as a fluorescence indicator for TDP-43 and ligand binding, leading to the discovery of A5 as a new TDP-43 ligand. **b.** Diagram of B3 fluorescence inhibition screening strategy. B3 (1 μM), tested compounds (A1-A5, 20 μM), and TDP-43 aggregates (6 μM) were incubated at room temperature for 1 h. The fluorescence intensity was measured and B3 fluorescence inhibition rate was calculated relative to compound-free controls, the inhibition rate of B3 fluorescence signals was calculated and normalized relative to the control group (without compound). **c.** B3 fluorescence inhibition assay results. Compounds A1-A5 reduced B3-TDP-43 fluorescence, with A5 exhibiting maximal inhibition. The data are the mean ± S.E.M., n = 3. Statistical significance was assessed using a one-way ANOVA followed by Dunnett's multiple comparisons test versus the control group. **d.** Validation of A5 by standard binding assays. A5 (20 μM) inhibited ThT (5 μM) fluorescence when interacting with 6 μM TDP-43 aggregates. The data are the mean ± S.E.M., n = 3, statistical significance was assessed using a one-way ANOVA followed by tukey's multiple comparisons test versus the Tht co-incubation with TDP-43 group. * $p < 0.05$, ***$p < 0.001$ **e.** MST binding affinity tests of NT-647-labeled TDP-43 in the presence of increasing concentrations of A5. The dissociation constant (K_d) of A5 for TDP-43 was calculated by plotting the fraction bound signal against A5 concentration, yielding an apparent K_d = 445 ± 141 nM (n = 3). These data confirmed the drug screening applications of B3 and identified A5 as a new TDP-43 ligand.

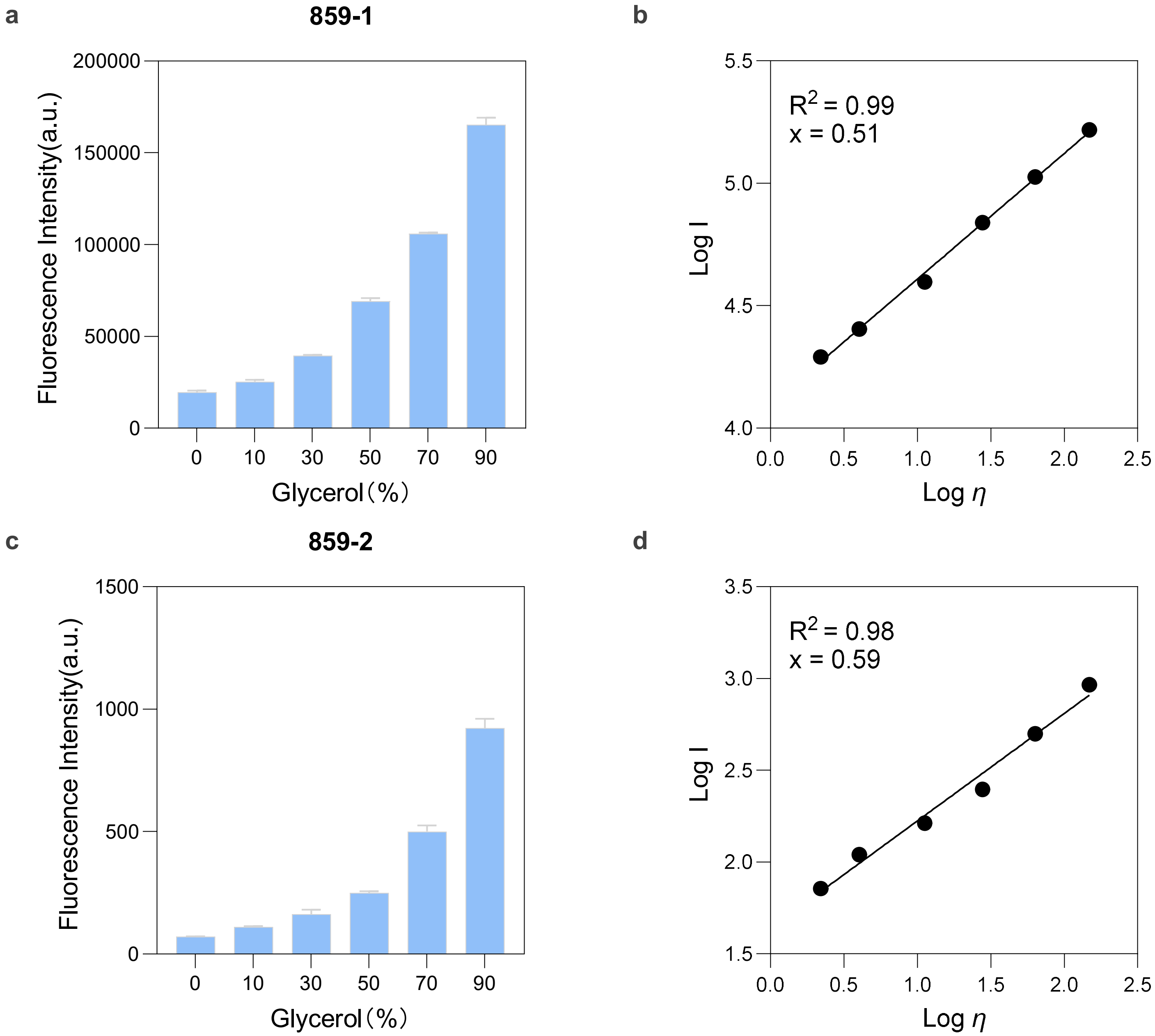

Extended Data Figure 9: **Fluorescence measurement in solution of increasing viscosity.** The fluorescence intensity of **859-1 (a)** and **859-2 (c)** was assessed in DMSO with varying glycerol percentages (0, 10, 30, 50, 70, 90%). Compounds were dissolved in the corresponding solvent mixtures, and fluorescence was measured on a microplate reader (n=3; 859-1: $\lambda ex = 539$ nm, $\lambda em = 610$ nm; 859-2: $\lambda ex = 590$ nm, $\lambda em = 643$ nm). Data are presented as mean ± S.E.M. from three independent experiments. Panels **(b, d)** show the linear relationship between the logarithm of fluorescence intensity (Log I) and the logarithm of viscosity (Log η) for **859-1** and **859-2**, respectively.

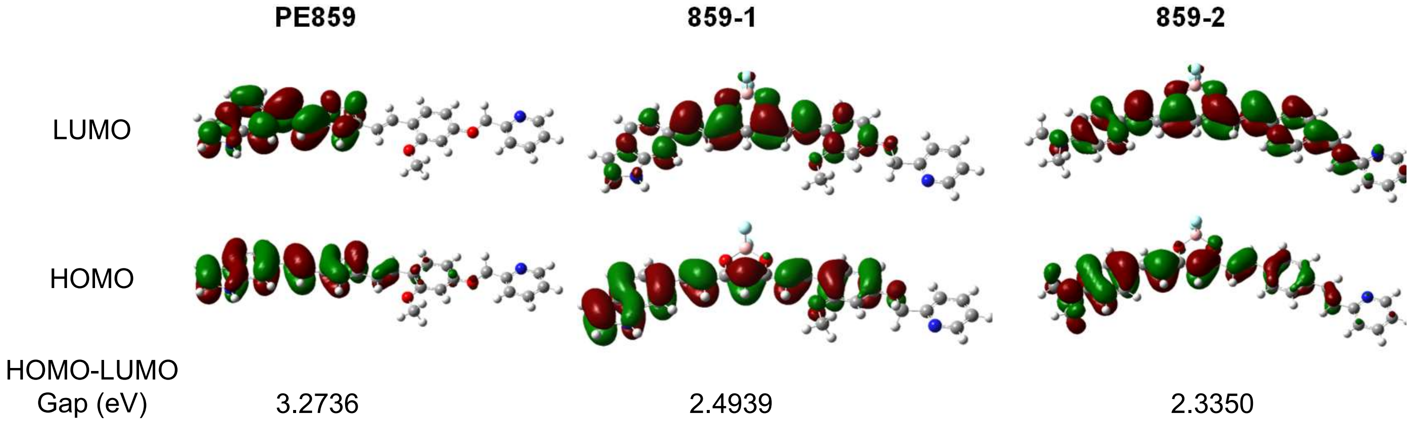

Extended Data Figure 10: **Calculated molecular frontier orbitals of PE859, 859-1 and 859-2.** HOMO (highest occupied molecular orbital); LUMO (Lowest unoccupied molecular orbital).

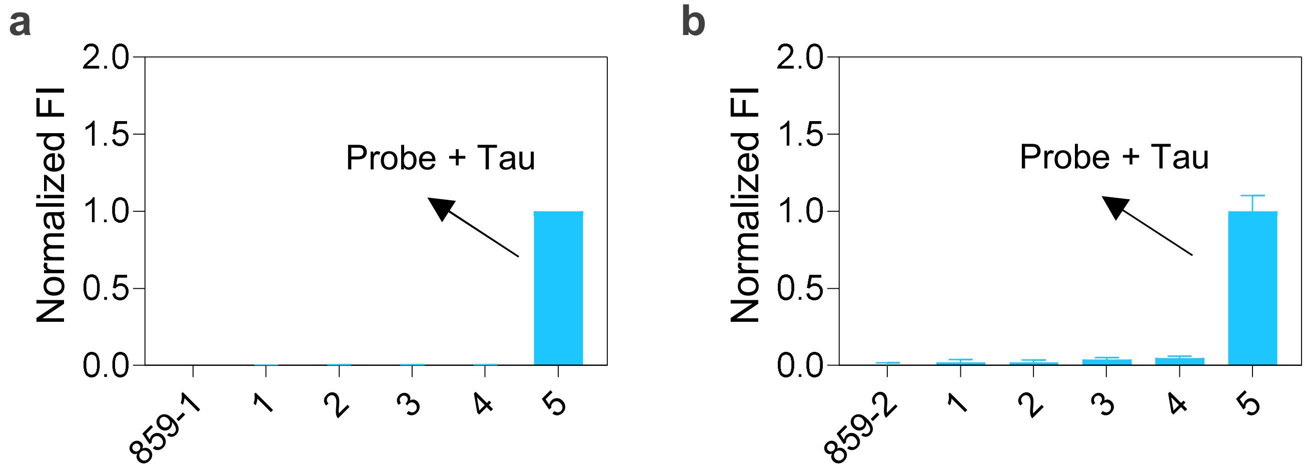

Extended Data Figure 11: **Selectivity assessment of candidate fluorescent probes.** **(a)** 859-1 and **(b)** 859-2 were mixed with various analytes (1. Trypsin, 2. HSA, 3. Lysozyme, 4. BSA, 5. Target (Tau aggregates, TDP-43 aggregates, or BCL-2 protein). Fluorescence intensity changes were recorded and normalized to the probe-only control. The results demonstrate that both probes exhibit negligible fluorescence response toward non-target proteins, confirming high binding specificity. The data are the mean ± S.E.M., n = 3.

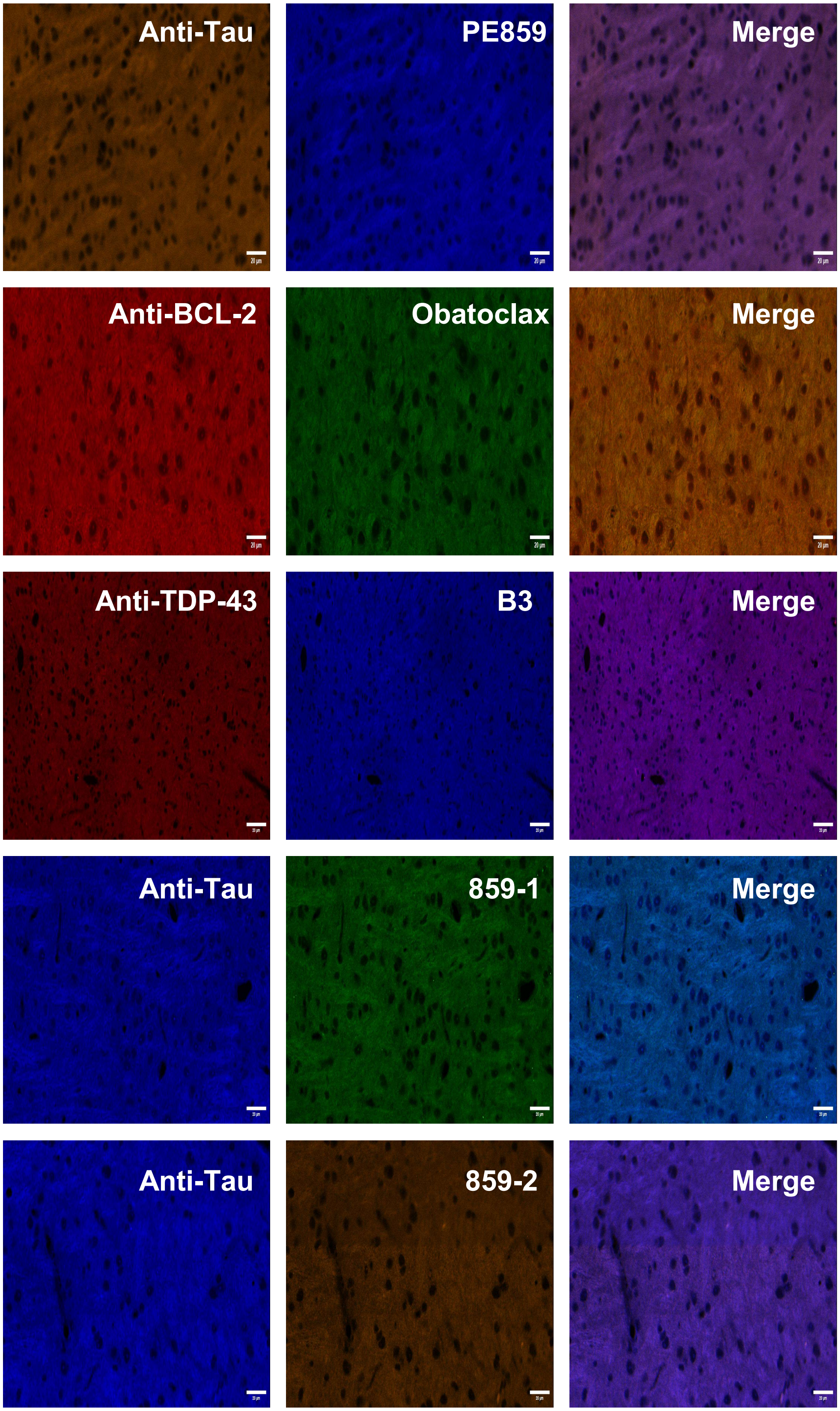

Extended Data Figure 12: **Fluorescence staining results of normal mice brain sections that endogenously lack the pathological targets.** **(a)** PE859, **(b)** obatoclax, **(c)** B3, **(d)** 859-1, and **(e)** 859-2. The sections were stained with the respective fluorophores and co-stained with target-specific antibodies. All probes produced only minimal background signal in these control sections. Scale bar: 20 μm.

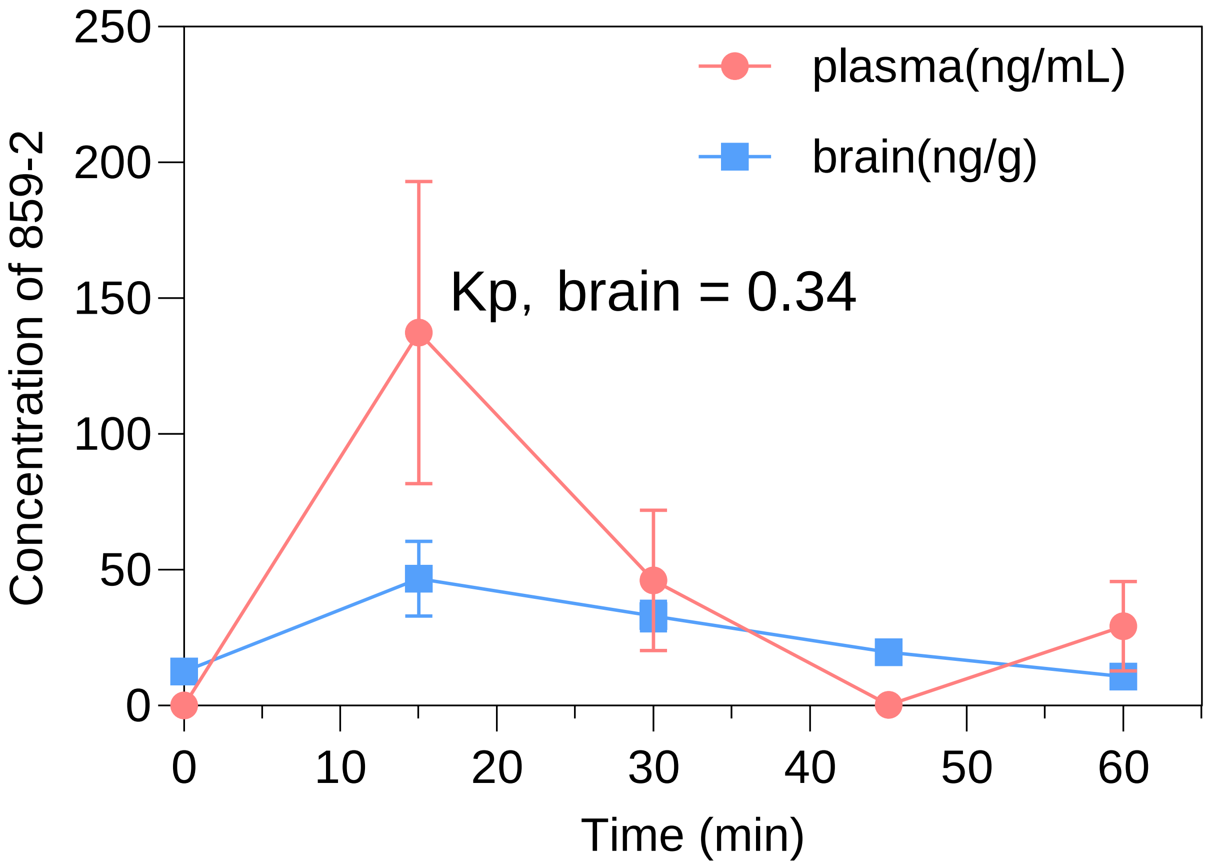

Extended Data Figure 13: **Pharmacokinetic profile and brain penetration of 859-2 in mice.** Plasma and brain concentrations of 859-2 were measured in female Balb/c mice (8 weeks old) over a 60-minute period following a single 4 mg/kg intravenous dose. Compound quantification was performed using LC-MS with a previously validated acetonitrile precipitation method. The resulting brain-to-plasma ratio (Kp,brain) was 0.34, indicating effective blood-brain barrier penetration. The data are the mean ± S.E.M., n = 3.

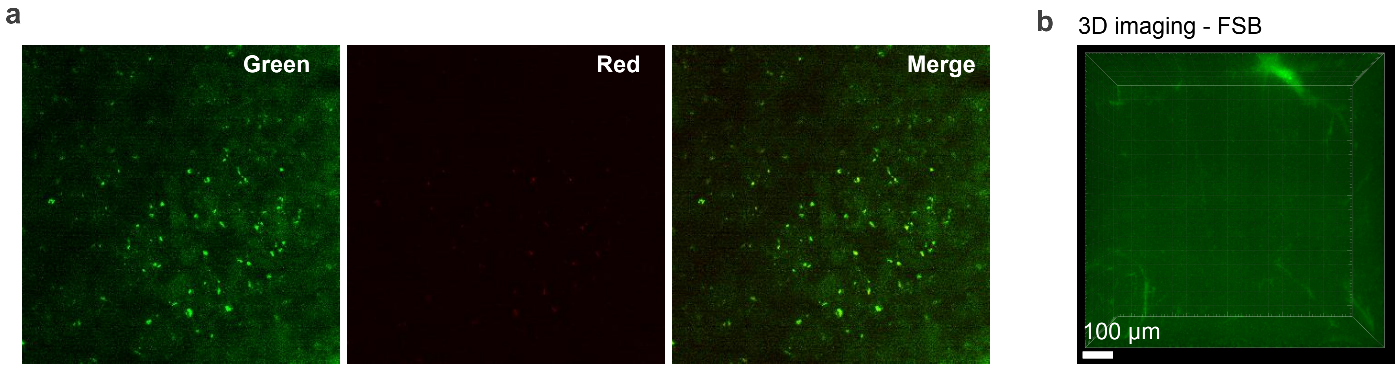

Extended Data Figure 14: **In vivo two-photon and 3D transparent tissue imaging of tau deposits using FSB. a)** In vivo two-photon imaging results of FSB only. FSB showed bright labeling of tau deposits in the green channel (800 nm) while weak signal in the red channel (1000 nm). These data exclude the interference of FSB in subsequent red-channel imaging using 859-2. **b)** 3D transparent brain imaging of FSB after tissue clearing process. FSB was unable to resist prolonged clearing process and its signals were almost lost.

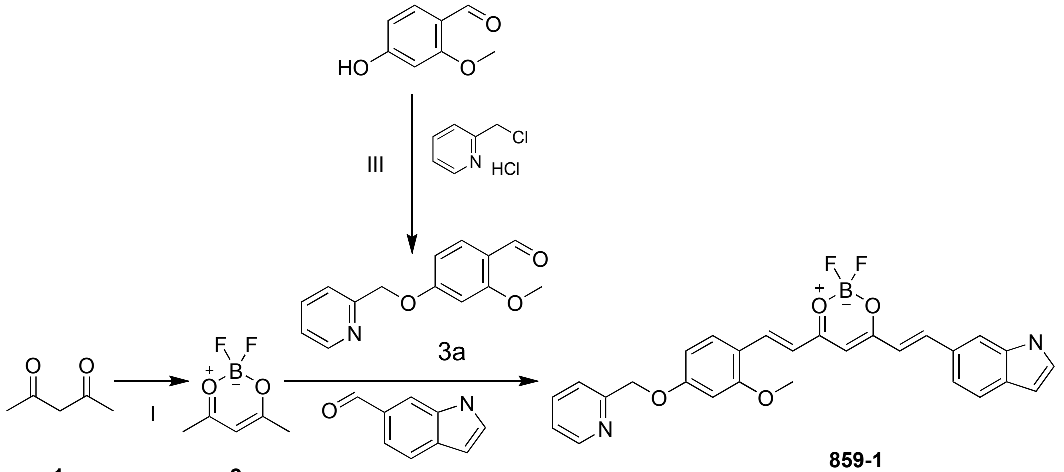

Extended Data Figure 15: **Synthetic route of 859-1.** Reagents and conditions: (I) K_2CO_3, $BF_3{\cdot}E_2O$, DCM; (II) tributyl borate, n-BuNH$_2$, Tol. (III) K_2CO_3, DMF.

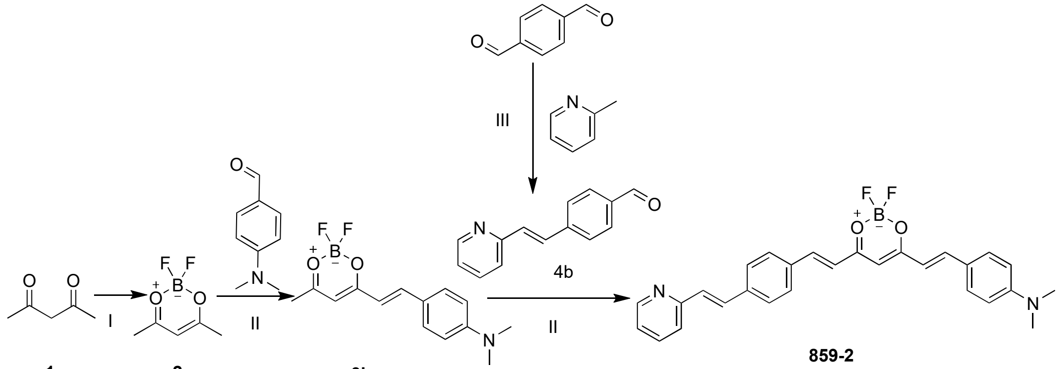

Extended Data Figure 16: **Synthetic route of 859-2.** Reagents and conditions: (I) K_2CO_3, $BF_3{\cdot}E_2O$, DCM; (II) n-BuNH$_2$, tributyl borate, Tol; (III) Ac_2O, AcOH.

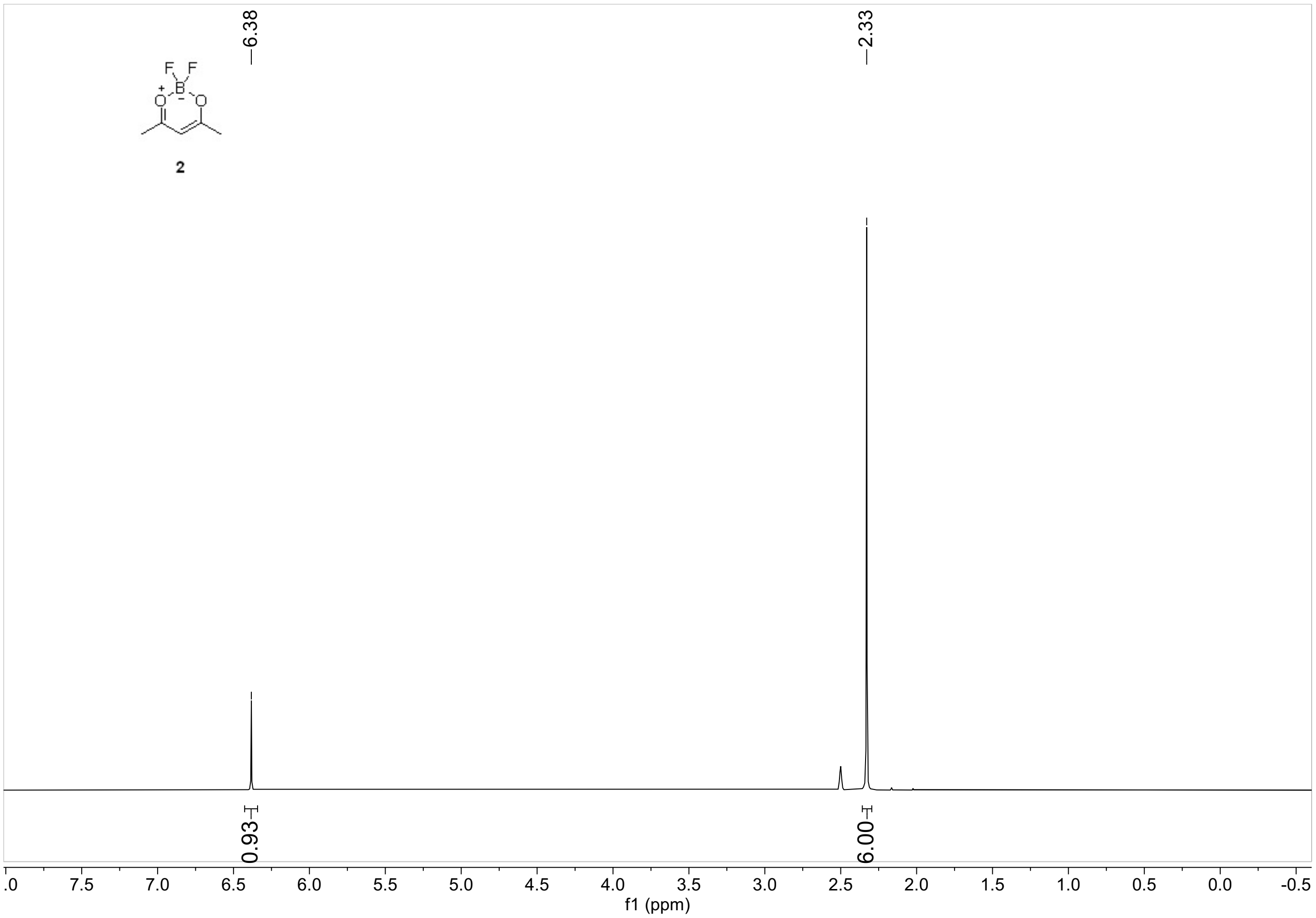

Extended Data Figure 17: ^{1}**H NMR spectra of 2 (400 MHz, DMSO-d6).** δ 6.38 (s, 1 H), 2.33 (s, 6 H).

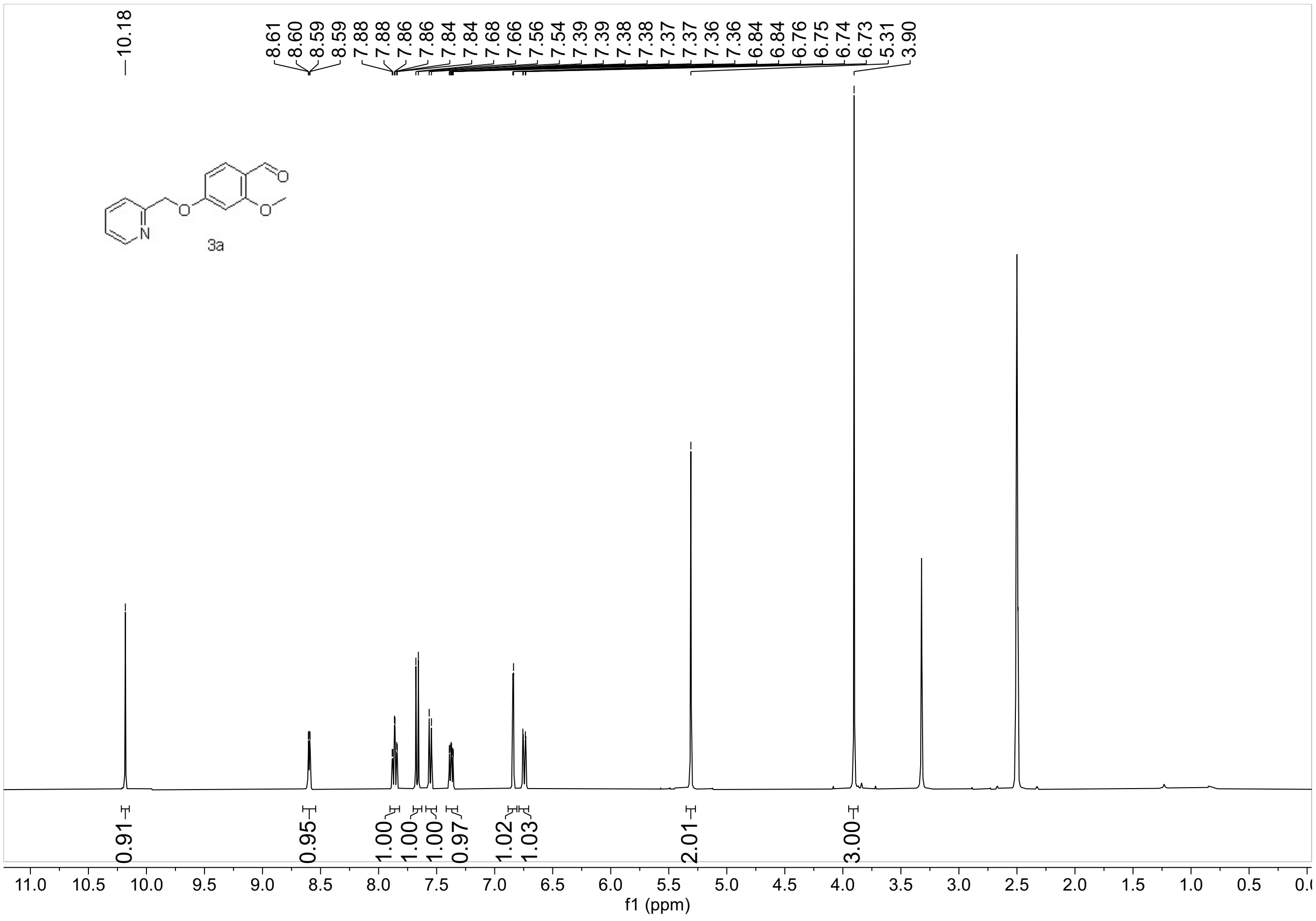

Extended Data Figure 18: [1]**H NMR spectra of 3a (400 MHz, DMSO-d_6).** δ 10.19 (s, 1 H), 8.58 - 8.63 (m, 1 H), 7.84-7.89 (m, 1 H), 7.67 (d, J = 8.7 Hz, 1 H), 7.56 (d, J = 7.8 Hz, 1 H), 7.35 - 7.41 (m, 1 H), 6.83 - 6.87 (m, 1 H), 6.70-6.80 (m, 1 H), 5.31 (s, 2 H), 3.91 (s, 3 H).

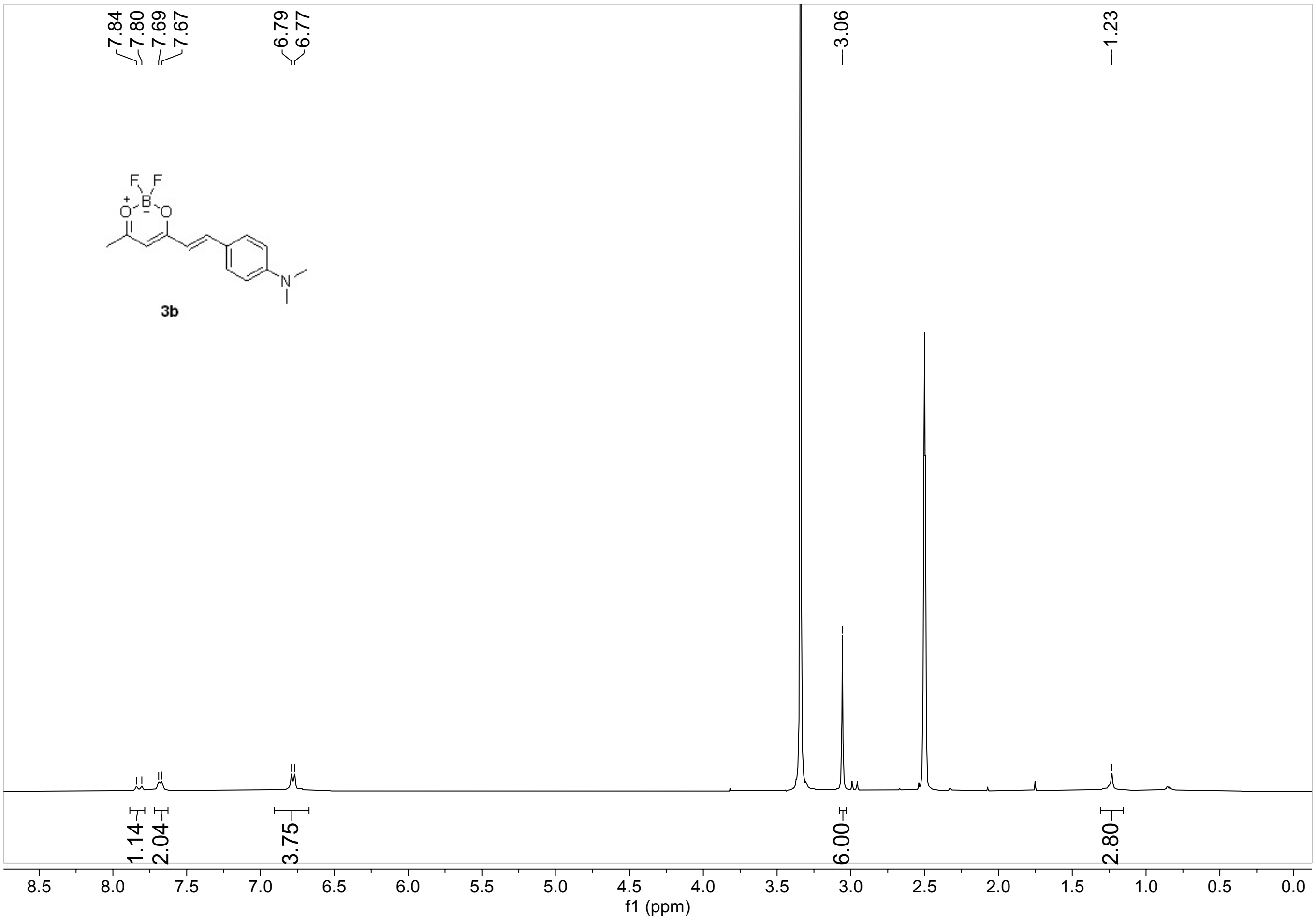

Extended Data Figure 19: ^{1}**H NMR spectra of 3b (400 MHz, DMSO-d_6).** δ ppm 7.82 (d, *J* = 14 Hz, 1 H), 7.68 (d, *J* = 7.6 Hz, 2 H), 6.67 - 6.89 (m, 4 H), 3.06 (s, 6 H), 1.13 - 1.33 (m, 3 H).

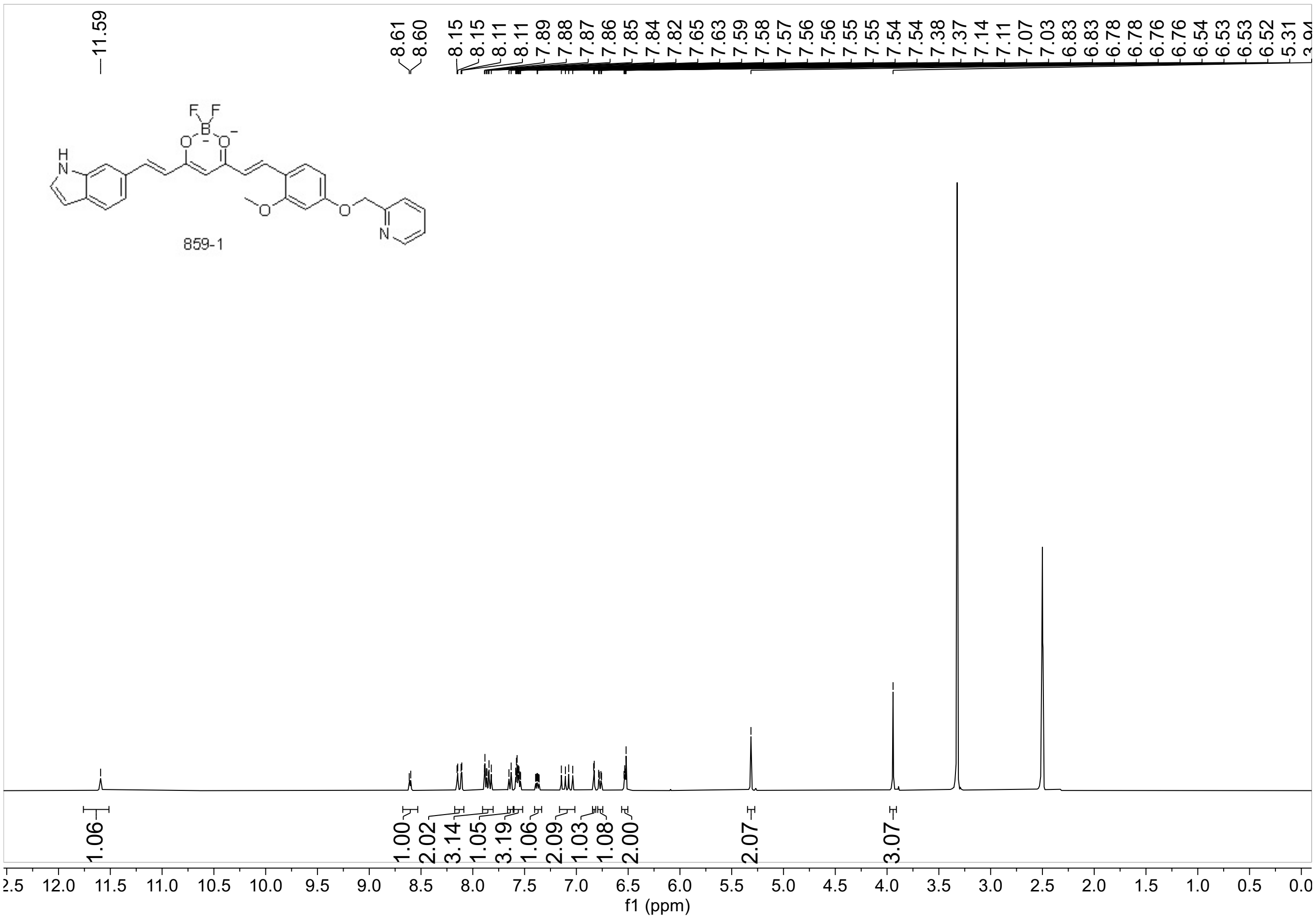

Extended Data Figure 20: [1]**H NMR spectra of 859-1 (400 MHz, DMSO-d_6).** δ ppm 11.60 (s, 1 H), 8.59 - 8.64 (m, 1 H), 8.14 (dd, *J* = 15.6, 2.6 Hz, 2 H), 7.82 - 7.91 (m, 3 H), 7.63 - 7.67 (m, 1 H), 7.54 - 7.60 (m, 3 H), 7.36 - 7.41 (m, 1 H), 7.03 - 7.16 (m, 2 H), 6.84 (d, *J* = 2.3 Hz, 1 H), 6.70-6.80 (m, 1 H), 6.52 - 6.56 (m, 2 H), 5.32 (s, 2 H), 3.95 (s, 3 H).

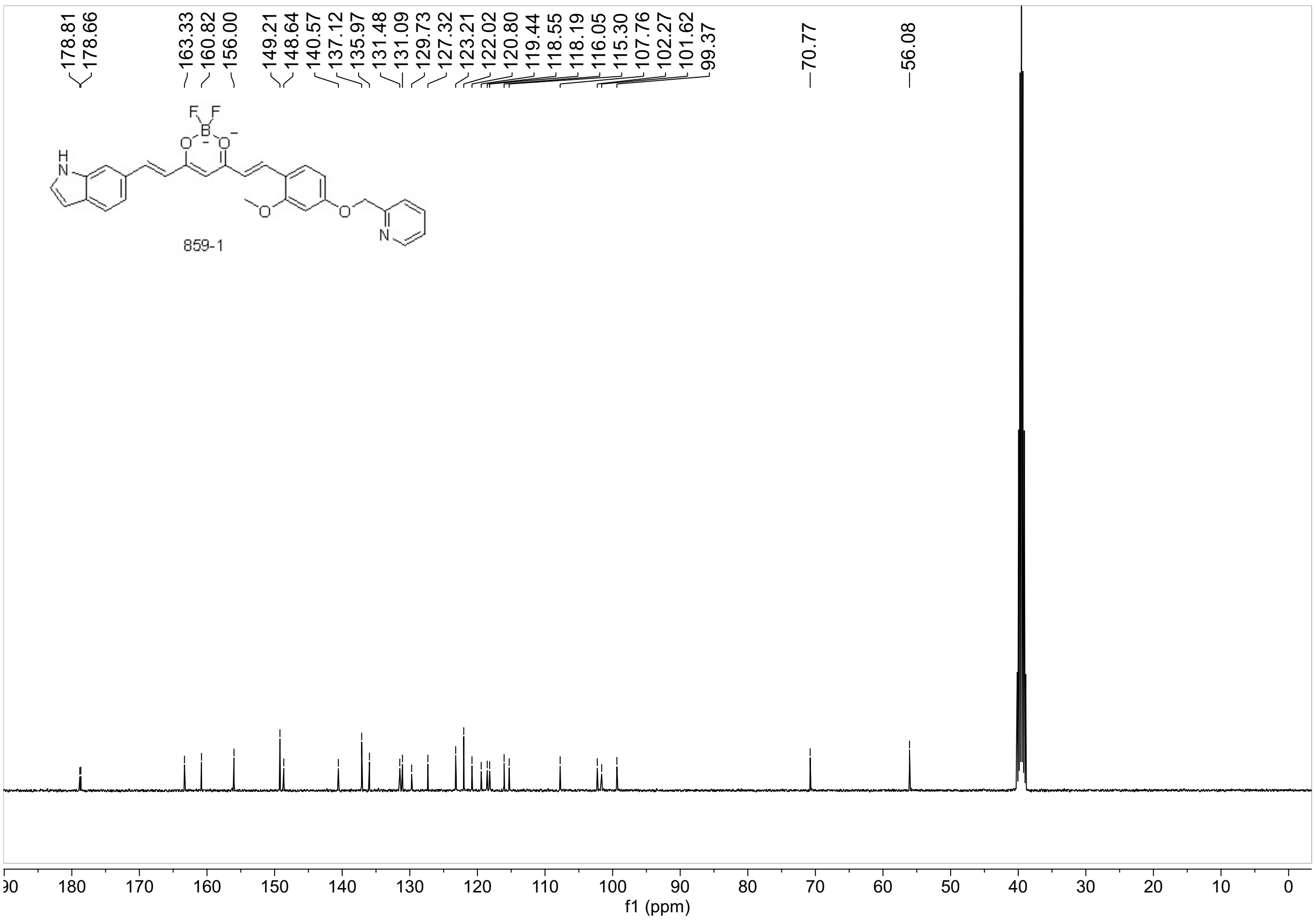

Extended Data Figure 21: **^{13}C NMR spectra of 859-1 (400 MHz, DMSO-d_6).** δ 179.272, 179.121, 163.796, 161.283, 156.466, 149.670, 149.103, 141.033, 137.584, 136.432, 131.944, 131.558, 130.189, 127.788, 123.675, 122.487, 121.267, 119.902, 119.010, 118.652, 116.515, 115.760, 108.224, 102.732, 102.082, 99.832, 71.234, 56.544..

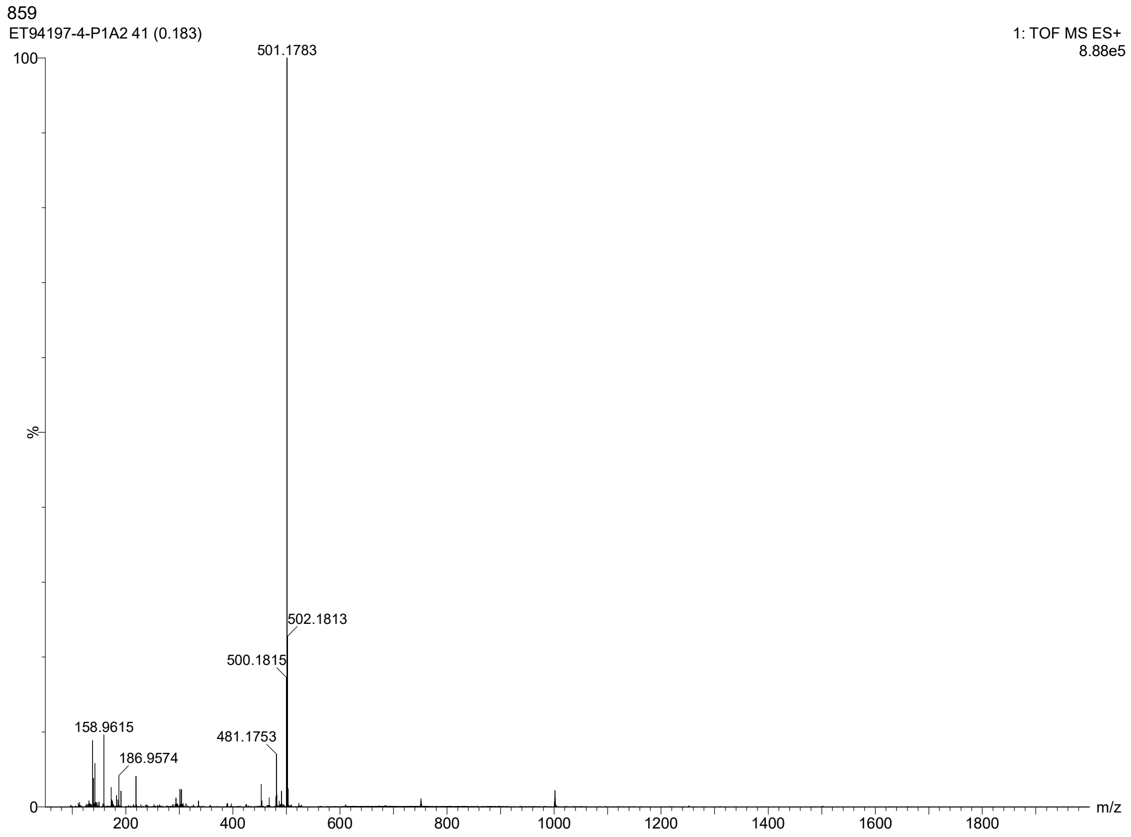

The test was performed in OPEN ACCESS R&D Laboratories. For research information only.

Extended Data Figure 22: **HRMS spectra of 859-1.** $[M+H]^+$ calc'd. for $C_{28}H_{24}BF_2N_2O_4$: 501.1797, found 501.1783.

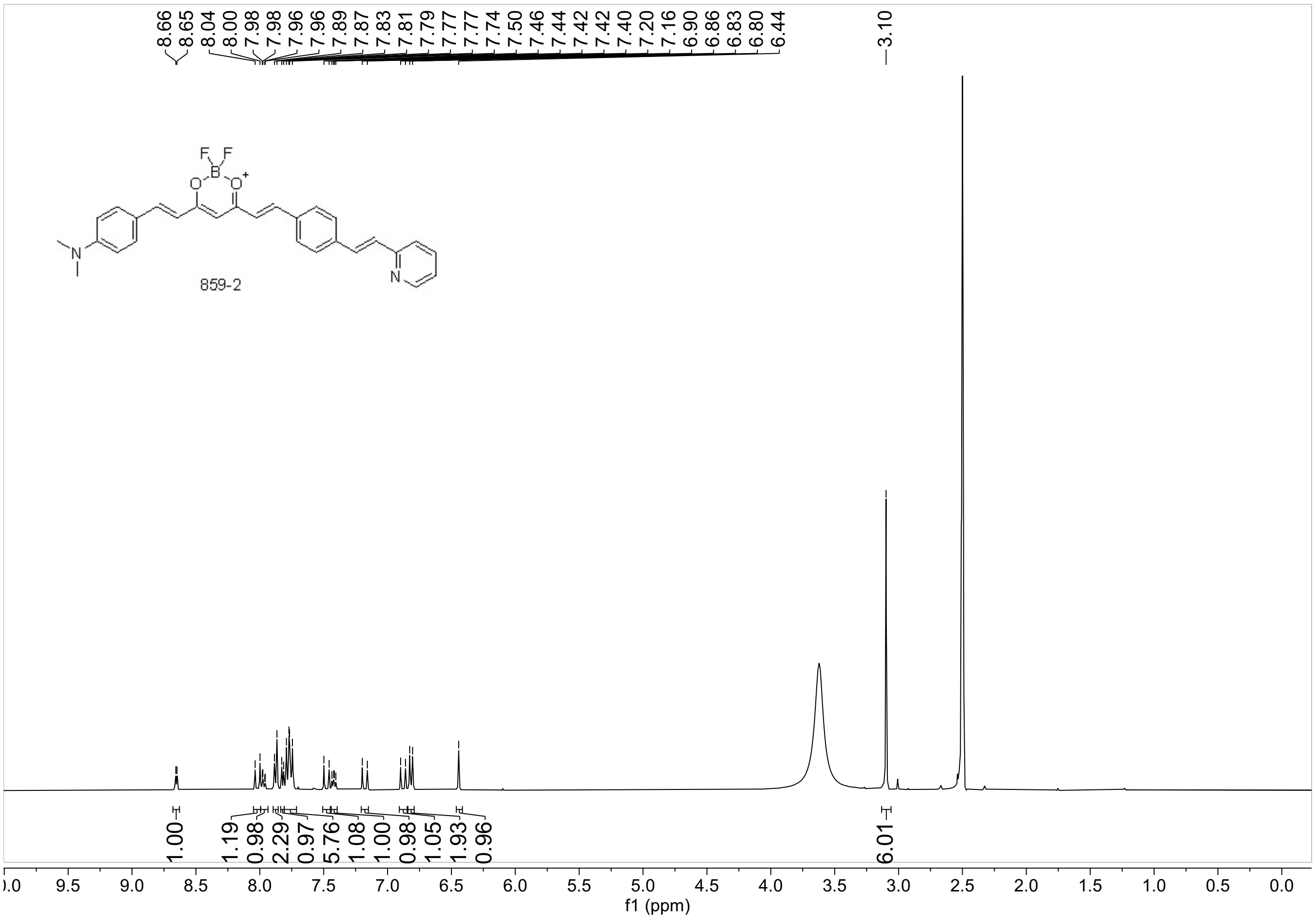

Extended Data Figure 23: [1]**H NMR spectra of 859-2 (400 MHz, DMSO-d_6).** δ ppm 8.63 - 8.68 (m, 1 H), 8.43 - 8.48 (m, 1 H), 8.24 - 8.29 (m, 1 H), 8.06 - 8.09 (m, 2 H), 7.81 - 7.91 (m, 1 H), 764 - 7.73 (m, 6 H), 7.44 - 7.49 (m, 1 H), 7.28 - 7.33 (m, 1 H), 7.07 - 7.16 (m, 1 H), 6.91 - 6.98 (m, 1 H), 6.72 - 6.77 (m, 2 H), 6.45 (s, 1 H), 3.09 (s, 6 H).

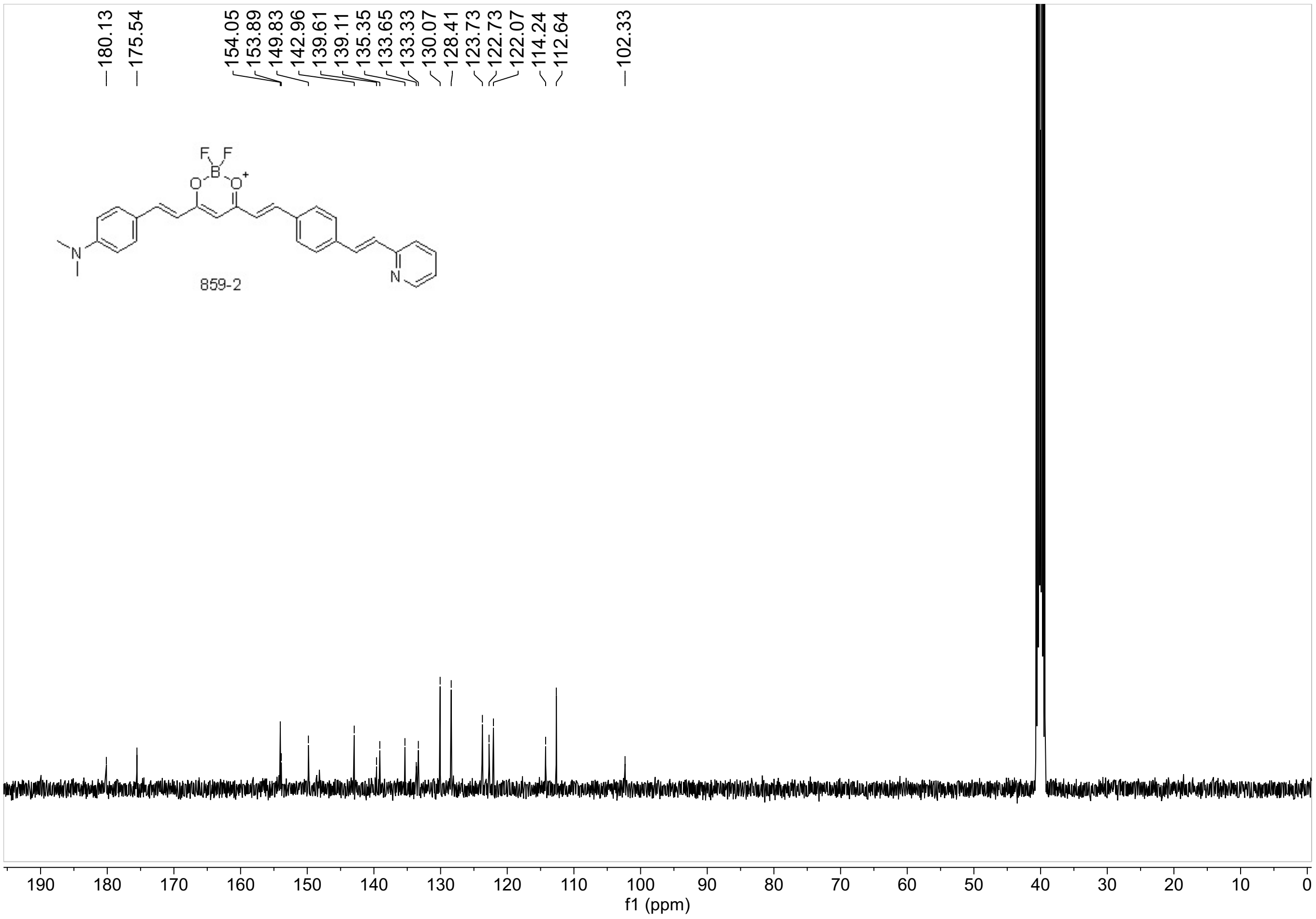

Extended Data Figure 24: **^{13}C NMR spectra of 859-2 (400 MHz, DMSO-d_6).** δ 180.122, 175.540, 154.041, 153.883, 149.824, 142.956, 139.605, 139.114, 135.345, 133.333, 130.066, 128.405, 123.783, 123.725, 122.732, 122.071, 114.243, 112.636, 102.324.

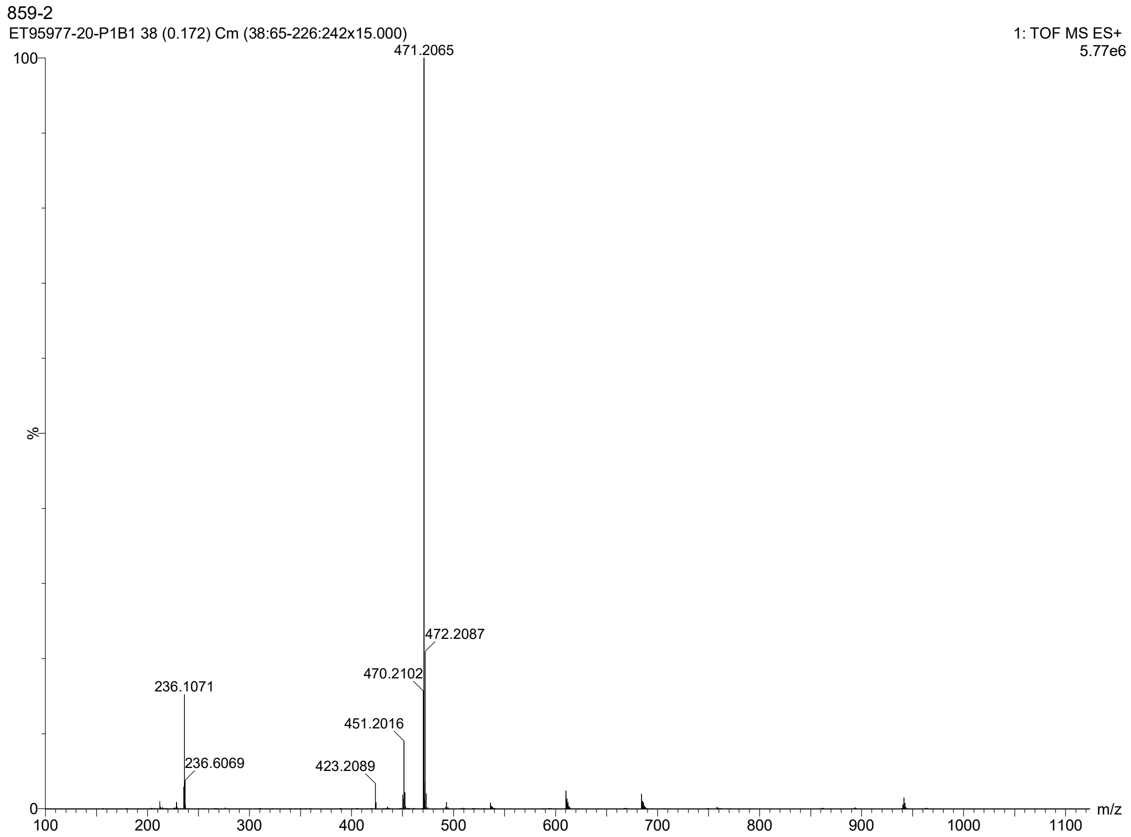

The test was performed in OPEN ACCESS R&D Laboratories. For research information only.

Extended Data Figure 25: **HRMS spectra of 859-2.** $[M+H]^+$ calc'd for $C_{28}H_{26}BF_2N_2O_2$ 471.2055, found.471.2065.

Extended Data Table 1: **Photophysical property of candidate molecules in acetonitrile (MeCN).** a: prediction data by PROBY-L1 and L2. b: Spectra assays data.

Compound ID	Target	λAbs^a(nm)	λEm^a (nm)	λAbs^b (nm)	λEm^b(nm)	ε
4852-0197(B1)	TDP-43	366	455	370	501	2400
2079-0021(B2)	TDP-43	367	461	342	455	21900
E201-0354(B3)	TDP-43	370	437	370	443	19600
E201-0140(B5)	TDP-43	366	469	382	450	19800
K505-0424(B6)	TDP-43	362	471	330	450	20200
T4077(PE859)	Tau	372	483	335	400	92780
T7003	Tau	361	497	655	672	96100
T6888	Tau	670	541	655	675	54800
T6778	BCL-2	620	627	633	656	14200
T6S0052	BCL-2	386	470	430	552	3600
T6275(Obatoclax)	BCL-2	567	615	480	540	38920

Extended Data Table 2: **Comparison of predicted and experimental extinction coefficient and quantum yield of fluorophores in MeCN.**

Compound	Data	$\log_{10} \varepsilon$	Quantum yield (PROBY^a/FLAME^b)
Obatoclax	predicted	4.3733	0.4994/0.2692
	experimental	4.5902	0.0069
859-1	predicted	4.8292	0.2406/0.3955
	experimental	4.9784	0.0063
859-2	predicted	4.8036	0.1342/0.1915
	experimental	4.8606	0.0105
B3	predicted	4.6563	0.3762/0.3373
	experimental	4.2923	0.2891
PE859	predicted	4.5870	0.2728/0.1940
	experimental	4.9675	0.1223

Extended Data Table 3: **Binding affinity of candidate probes.**

candidates	target	Trysin	HSA	Lysozyme	BSA
PE859	0.66 μM (Tau)	n.d.	n.d.	n.d.	n.d.
B3	1.122 μM (TDP-43)	n.d.	n.d.	n.d.	n.d.
obatoclax	220 nM (BCL-2)	n.d.	n.d.	n.d.	n.d.
859-1	216.8 nM (Tau)	n.d.	n.d.	n.d.	n.d.
859-2	54.8 nM (Tau)	n.d.	n.d.	n.d.	n.d.

n.d. = not detected (signal was below the reliable detection limit of the assay)

Extended Data Table 4: **Docking scores of top 30 candidate compounds for TDP-43 protein.**

IDNUMBER	logP	docking score	MMGBSA dG Bind	mol MW	IFDScore
Y600-4165	2.42	-3.448	-36.46	396.531	-157.3
SB91-0886	2.34	-6.708	-35.91	333.448	-159.92
D665-0187	4.38	-4.001	-31.01	314.211	-157.57
SB95-0978	2.3	-5.19	-37.96	354.494	-157.16
F360-0426	4.38	-4.057	-34.39	319.793	-156.35
Y043-2803	2.03	-5.355	-30.24	317.362	-156.95
M575-0559	3.407	-5.617	-31.24	374.842	-160.7
J032-0080	3.97	-4.261	-31.04	311.355	-157.98
P087-2841	2.42	-3.491	-36.18	381.449	-158.12
D030-0213	4.24	-4.069	-34.71	378.488	-158.39
5692-1201	4.68	-4.505	-32.89	350.367	-158
SC12-0732	2.6	-5.268	-32.76	364.459	-159.29
CM4146-1334	2.69	-4.716	-31.42	396.531	-159.44
G396-0987	4.71	-3.894	-30.58	412.915	-158.15
E677-0478	4.67	-3.243	-31.57	385.498	-156.83
E146-1331	2.36	-3.82	-31.51	328.413	-156.12
V028-3669	3.27	-4.915	-40.63	407.555	-161.13
J077-1268	2.83	-5.391	-37.56	414.934	-157.78
Y207-3819	3.44	-3.985	-31.65	282.385	-156.61
SB58-0938	3.31	-3.588	-31.31	425.957	-158.15
S823-4333	2.5	-4.728	-31.16	332.322	-158
V030-4110	2.13	-3.735	-33.1	299.375	-154.36
S664-5056	2.58	-5.228	-35.91	312.411	-163.52
8013-3623	3.94	-4.306	-36.73	373.161	-162.11
SD25-0025	2.42	-5.047	-31.28	287.404	-158.63
E641-0523	2.79	-4.075	-31.78	355.479	-157.76
S858-2379	2.33	-4.17	-30.95	340.421	-156.94
4964-5043	3.02	-3.274	-33.69	301.341	-155.62
ZC44-0005	3.24	-4.547	-35.1	355.482	-159.19
P201-0747	2.516	-3.789	-35.34	309.367	-158.77

Extended Data Table 5: **Photophysical property of PE859, 859-1 and 859-2 in various solvents.**

Compound	Solvent	Viscosity (cP)	Dielectric constant(ε)	Solvent Polarizability	Solvent Dipolarity	λAbs^a(nm)	λ Em(nm)
PE859	H2O	1	78.5	0.681	0.997	352	422
	MeOH	0.55	32.6	0.608	0.904	337	410
	EtOH	1.1	24.55	0.633	0.783	335	397
	DMSO	1.996	48.9	0.830	1.000	343	408
	MeCN	0.37	37.5	0.645	0.974	338	402
859-1	H2O	1	78.5	0.681	0.997	510	-
	MeOH	0.55	32.6	0.608	0.904	514	595
	EtOH	1.1	24.55	0.633	0.783	513	588
	DMSO	1.996	48.9	0.830	1.000	539	610
	MeCN	0.37	37.5	0.645	0.974	514	594
859-2	H2O	1	78.5	0.681	0.997	550	630
	MeOH	0.55	32.6	0.608	0.904	562	641
	EtOH	1.1	24.55	0.633	0.783	560	648
	DMSO	1.996	48.9	0.830	1.000	590	643
	MeCN	0.37	37.5	0.645	0.974	570	662

Extended Data Table 6: **Dissociation constant (K_d) and docking score of optimized probes with tau protein.**

Probe name	K_d (tau)	Docking score
859-1	216.8 ± 265.4 nM	-5.033
859-2	54.8 ± 38.5 nM	-6.730
